# Brain hierarchy score: Which deep neural networks are hierarchically brain-like?

**DOI:** 10.1016/j.isci.2021.103013

**Published:** 2021-08-21

**Authors:** Soma Nonaka, Kei Majima, Shuntaro C. Aoki, Yukiyasu Kamitani

**Affiliations:** 1 Faculty of Integrated Human Studies, Kyoto University, Kyoto 606-8501, Japan; 2 Graduate School of Informatics, Kyoto University, Kyoto 606-8501, Japan; 3 ATR Computational Neuroscience Laboratories, Seika, Kyoto 619-0288, Japan

**Keywords:** Neuroscience, Neural networks, Human-centered computing

## Abstract

Achievement of human-level image recognition by deep neural networks (DNNs) has spurred interest in whether and how DNNs are brain-like. Both DNNs and the visual cortex perform hierarchical processing, and correspondence has been shown between hierarchical visual areas and DNN layers in representing visual features. Here, we propose the brain hierarchy (BH) score as a metric to quantify the degree of hierarchical correspondence based on neural decoding and encoding analyses where DNN unit activations and human brain activity are predicted from each other. We find that BH scores for 29 pre-trained DNNs with various architectures are negatively correlated with image recognition performance, thus indicating that recently developed high-performance DNNs are not necessarily brain-like. Experimental manipulations of DNN models suggest that single-path sequential feedforward architecture with broad spatial integration is critical to brain-like hierarchy. Our method may provide new ways to design DNNs in light of their representational homology to the brain.

## Highlights

• A measure for brain-like hierarchy is proposed to characterize DNNs
• Encoding/decoding with human fMRI quantifies the hierarchical correspondence
• Among representative DNN models, high-performance models are *not* brain-like
• Critical factors for brain-like hierarchy are explored

## Graphical abstract

**Figure:**
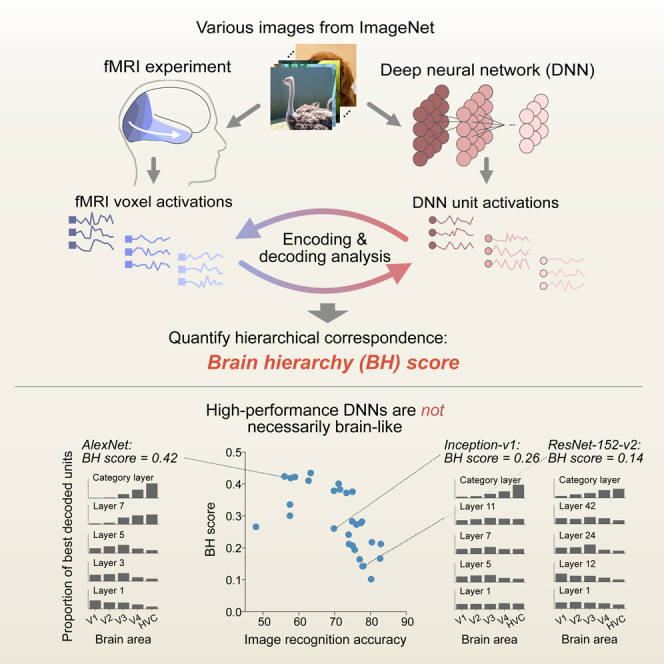

## Introduction

The design of deep neural networks (DNNs) is typically based on brain-like multi-stage hierarchical structures. DNNs have been reported to achieve human-level performance in some cognitive tasks, including image recognition (Krizhevsky et al., 2012). The structural and behavioral similarities between DNNs and biological brains have triggered investigation on how DNNs and biological brains are “functionally” similar. A large number of studies have suggested that task-optimized DNNs acquire similar representations to task-related brain regions. For example, neuronal responses in the inferior temporal (IT) cortex of monkeys, which underlies object recognition, are predicted accurately by unit activation patterns of DNNs trained to perform visual tasks (Cadieu et al., 2014; Khaligh-Razavi and Kriegeskorte, 2014; Yamins et al., 2014). DNN's activations can also successfully explain human functional magnetic resonance imaging (fMRI) responses (Eickenberg et al., 2017; Guclu and van Gerven, 2015; Khaligh-Razavi and Kriegeskorte, 2014). Moreover, recent studies have demonstrated hierarchical correspondence of representations or hierarchical homology between DNNs and biological brains (Cichy et al., 2016; Guclu and van Gerven, 2015; Horikawa and Kamitani, 2017). Horikawa and Kamitani (2017) reported that human fMRI responses to visual images can be decoded (translated) into unit activations of a DNN (AlexNet) responding to the same images. The brain areas that best predict unit activations in a DNN layer are reported to have gradually shifted from lower (e.g., V1, V2, and V3) to middle and higher visual areas (e.g., V4, lateral occipital complex, fusiform face area, and parahippocampal place area) as the target DNN layer shifts from lower to higher. This finding indicates the functional similarity of the hierarchical representations between DNNs and biological brains.

Why is the functional similarity between DNNs and the brain important? Brain-like hierarchical representations have the potential to realize DNNs that exhibit more human-like behavior, and to overcome the limitations of the current task-optimized DNNs. To design and train DNNs to develop hierarchical representations similar to the human brain, DNNs are expected to share the feature spaces with the human brain and emulate its internal information processing. Such DNNs would behave more similarly to humans than DNNs trained to maximize the task performance. Potential advantages of the brain-like DNNs include robustness against adversarial attack (Szegedy et al., 2014), generalizability across datasets with various types of image distortion (Geirhos et al., 2019), and realistic behavior patterns as surrogates of humans. In addition to the potential benefits of the development of DNNs, brain–DNN functional similarity measurement may advance our understanding of the brain by providing better computational models (Yamins and DiCarlo, 2016) or experimental tools generating optimal stimuli for neurons (Bashivan et al., 2019; Ponce et al., 2019). Thus, quantitative measures for evaluating how a given DNN has similar representations to the brain and how the representations develop brain-like hierarchies may have a range of valuable applications.

In a previous study (Schrimpf et al., 2018), the Brain-Score was proposed as a framework to quantitatively measure the brain–DNN similarity of representations. The Brain-Score evaluates how accurately neuronal responses in primate visual areas were predicted from DNN unit activation patterns. This previous study systematically compared the similarities between various DNNs for image recognition and representations in middle and higher visual areas (V4 and IT cortex), reporting a positive correlation between object classification accuracy on the ImageNet dataset (i.e., ImageNet top-1 accuracy) and Brain-Score across 69 DNNs. However, this correlation was absent for more recently developed high-performance DNNs (DNNs with ≥70% accuracy), suggesting that performance improvement does not necessarily lead to brain-like DNNs. Although the Brain-Score captures the similarity of representations between DNNs and individual brain areas, it does not evaluate hierarchical homology across layers/brain areas between DNNs and the brain. Because multistage hierarchical processing is considered to play a vital role in perception and cognition, quantitative evaluation of the brain–DNN hierarchical homology may provide a sensitive comparison of functional similarity between DNNs and the human brain.

**Figure 1:**
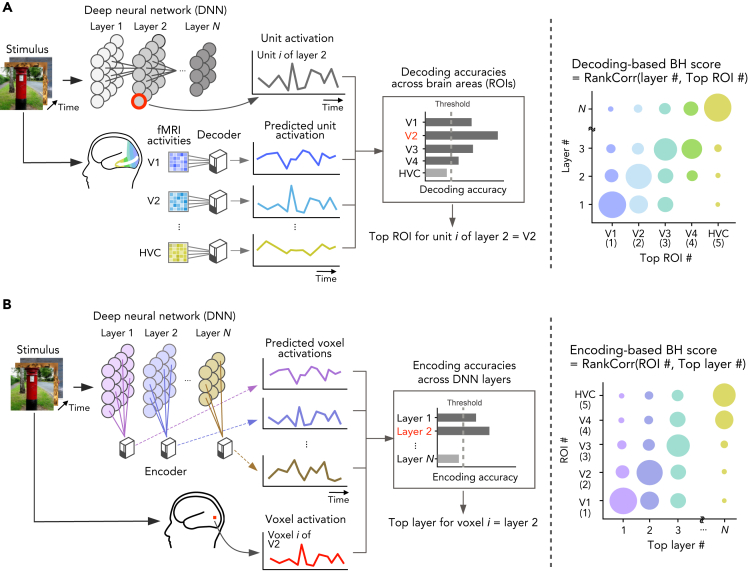
Evaluation of hierarchical homology (A) Decoding-based brain hierarchy (BH) score. To characterize individual deep neural network (DNN) units using human functional magnetic resonance imaging (fMRI), the responses of each DNN unit given natural images were predicted (decoded) from the fMRI responses to the same images in each of five ROI in the visual cortex. The ROI showing the best decoding accuracy (top ROI) was assigned to the unit (left). The decoding-based BH score was evaluated using the Spearman rank correlation between the layer and the top ROI numbers (right). When DNNs to be compared have different number of layers, we randomly subsampled five layers, and calculated the mean BH score across random selections. The units that were not predicted by any of the ROIs above a statistical threshold were excluded from the computation of the BH score. (B) Encoding-based BH score. We defined encoding-based BH score by exchanging DNN units and fMRI voxels. We predicted each fMRI voxel responses from activities of DNN units given natural images. The layer showing the best encoding accuracy (top layer) was assigned to the fMRI voxel (left). The encoding-based BH score was defined by the Spearman rank correlation between the ROI and top layer numbers (right).

In the current study, to quantitatively evaluate the degree of brain–DNN hierarchical homology across DNN layers and cortical areas, we propose a metric called the brain hierarchy (BH) score. The BH score is designed to capture the extent to which DNN layers of a given DNN are aligned with the hierarchy of the brain. To evaluate the hierarchical correspondence between DNN layers and brain regions, we used decoding and encoding approaches. To compute the decoding-based BH score, we characterize individual DNN units by decoding analysis where unit activations of DNNs are predicted from brain activity (Horikawa and Kamitani, 2017). We predict the activation of each unit from fMRI responses in one of five brain areas (regions of interest; ROIs) covering the ventral visual pathway (V1, V2, V3, V4, and higher visual cortex [HVC]), then identify the ROI showing the highest decoding accuracy among the five ROIs (hereafter referred to as the “top ROI”; Figure 1A). The decoding-based BH score is defined as the Spearman rank correlation coefficient between the layer number and the top ROI across units in the given DNN (Figure 1A). When the top ROIs of DNN units monotonically increase with respect to their layer number in a given DNN, the DNN shows the highest score. As a complementary measure, by exchanging DNN layers and brain areas, the encoding-based BH score is also calculated based on encoding models that predict fMRI voxel values from DNN unit activities (Figure 1B). Unless stated otherwise, the average of those two measures is reported as the BH score. Although many previous studies relied on the encoding approach to detect brain–DNN similarities, the decoding approach provides a complementary evaluation and allows for straightforward characterizations of individual units in a DNN by explaining/predicting the unit activations by brain activity patterns. Thus, we discuss the hierarchical correspondence in each DNN by looking at how the distribution of each DNN unit's top ROI shifts across the hierarchical layers.

Using the BH score and the distributions of the top ROIs for DNN units in each layer, we examine the degree of hierarchical similarity to the brain in 29 representative DNNs that were pre-trained on an object classification task, including AlexNet, the VGG family, the ResNet family, the DenseNet family, and the Inception family (see STAR Methods: “Deep neural networks” and Table 1). Note that, unlike Schrimpf et al. (2018), we focused on DNNs with high performance in the ImageNet large scale visual recognition challenge (more than 70% accuracy). DNNs with the same architectures and random weights are also studied to examine the effects of training. We investigate the correlations between BH scores and ImageNet top-1 accuracy to determine whether high-performance DNNs are hierarchically more brain-like, while testing the robustness of the BH score and the relationship with other measures of brain–DNN similarity.

**Table 1:** 29 deep neural networks compared in the current study

Model	ImageNet top-1 accuracy (%)	Full-connection	Skip-connection	Branch-connection	Depth	# Of weight parameters
AlexNet	57	✓	–	–	8	61M
VGG-16	71	✓	–	–	16	138M
VGG-19	72	✓	–	–	19	143M
VGG-F	59	✓	–	–	8	61M
VGG-M	63	✓	–	–	8	103M
VGG-S	63	✓	–	–	8	103M
ResNet-18	70	–	✓	–	18	12M
ResNet-34	73	–	✓	–	34	22M
ResNet-50-v2	76	–	✓	–	50	26M
ResNet-101-v2	77	–	✓	–	101	45M
ResNet152-v2	78	–	✓	–	152	60M
DenseNet-121Net-121	75	–	✓	–	121	8M
DenseNet-161	78	–	✓	–	161	29M
DenseNet-169	76	–	✓	–	169	14M
DenseNet-201	77	–	✓	–	201	20M
Inception-v1	70	–	–	✓	22	6M
Inception-v2	74	–	–	✓	34	11M
Inception-v3	78	–	–	✓	48	27M
Inception-v4	80	–	–	✓	76	46M
Inception-ResNet-v2	80	–	✓	✓	136	59M
CORnet-Z	48	–	–	–	5	2M
CORnet-R	56	–	–	–	9	5M
CORnet-S	75	–	✓	–	12	53M
SqueezeNet-1.0	58	–	–	–	18	1M
SqueezeNet-1.1	58	–	–	–	18	1M
MobileNet-v2-1.4-224	75	–	✓	–	51	7M
NASNet-Mobile	74	–	✓	✓	34	5M
NASNet-Large	83	–	✓	✓	46	89M
PNASNet-Large	83	–	✓	✓	46	86M

We then seek to identify architectural characteristics of DNNs associated with the degree of hierarchical homology. We focus on five representative components of DNN architecture: the presence of fully-connected (FC) layers, the presence of branch-connections, the presence of skip-connections, the number of DNN layers, and the number of weight parameters. These architectural components are compared with BH scores among the 29 pre-trained DNNs. Then, some of the components are experimentally manipulated to evaluate the effects on BH scores in trained DNNs with the rest of the architectures being identical. The code and the fMRI data to compute the BH score for novel DNNs are provided for public use at repositories (see STAR Methods: “Data and code availability”).

## Results

### Brain hierarchy score

The brain hierarchy (BH) score is designed to evaluate a deep neural network (DNN) for object recognition in terms of the hierarchical similarity to the human brain by determining the correspondence between DNN layers and visual cortical areas (regions of interests, ROIs) through decoding and encoding analyses. To consider hierarchical representations of DNNs, we included representative layers of each DNN in the analysis: the first layer, the last fully-connected (FC) layer (referred to as the “category layer” in the current study), the other FC layers, convolutional layers that do not belong to submodules (i.e., convolutional-block, skip-block, or branch-block), and the output layers of submodules. To treat DNN layers with and without submodules in a consistent manner, we used the output layers of submodules and excluded the internal layers inside those submodules in the computation of the BH score. Hereafter, “layer” refers to the representative layer, unless otherwise stated. The layers in each DNN are numbered from input to output. Each DNN unit is labeled by the layer number it belongs to. The brain ROIs include V1, V2, V3, V4, and a combined region of ventral object-responsive areas (higher visual cortex [HVC]; see STAR Methods: “Region of interest”), and each is assigned an ROI number: V1 (1), V2 (2), V3 (3), V4 (4), and HVC (5).

Here we describe in detail how to calculate BH scores based on decoding of DNN's unit activation from fMRI signal patterns in each brain area (decoding-based BH score; Figure 1A). The encoding-based BH scores were obtained by the same procedure but swapping units in the DNN and voxels in the fMRI data. For each DNN unit, we identified the ROI with the best linear decodability (i.e., the ROI whose functional magnetic resonance imaging [fMRI] voxel pattern can best predict the DNN unit activity using a linear decoder given the same input image) which we refer to as the “top ROI.” DNN units that were poorly predicted from any of the ROIs were excluded in later analyses to improve sensitivity of the BH score to the hierarchical representations (see STAR Methods: “Brain hierarchy score” for details). For the first DNN layer in which linear spatial filtering is performed, the absolute values of the raw unit activations were used as targets for decoding, because fMRI signals are known to be sensitive to deviations from baseline luminance but not luminance itself, even at V1, presumably reflecting complex cell-like early nonlinear processing (Haynes et al., 2004; see STAR Methods: “Decoding analysis”). We used the unit selection and the first layer nonlinearity as default settings. We discuss how these settings affected BH scores in a later section. Repeating the procedures for the DNN units used in the decoding analysis, we obtained the distributions of the top ROIs for each layer for visualization (Figure S1). If a DNN has hierarchical representations that are similar to those of the brain, the distribution of top ROIs should gradually shift from lower to higher cortical areas as the DNN layer increases.

The decoding-based BH score quantifies this gradual shift of top ROIs, exhibiting a high value if the distribution monotonically shifts with the layer number, and a low value if 1) the peak does not monotonically shift or 2) the distribution is flat or multimodal with highly variable top ROIs at each layer. Because the number of layers differs across DNNs and brain ROIs could be delineated differently, a linear relationship would not be expected between the layer and ROI numbers. Thus, we used the Spearman rank correlation between the layer and ROI numbers, measuring the degree of the monotonic relationship between the two variables.

The decoding-based BH score is defined by the Spearman rank correlation coefficient between the layer number and the top ROI number across DNN units. To compute the score, we sorted the values of variables in descending order and assign their positions in the rankings to them. When tied values (i.e., identical rank position) were present in the data, their mean position was assigned. The assigned values are called the fractional ranks. In this study, we denote the fractional ranks of the layer number and the top ROI number of the *i*-th DNN unit by $f_{i}^{\text{Layer}}$ and $f_{i}^{\text{ROI}}\;\left(i=1,\ldots ,I\right)$, respectively. The score was then computed using the Spearman rank correlation coefficient between the layer number and the top ROI number, which is expressed using their fractional ranks as follows:

$$\text{BH score}=\frac{\sum_{i=1}^{I}\left(f_{i}^{\text{Layer}}-\mu^{\text{Layer}}\right)\left(f_{i}^{\text{ROI}}-\mu^{\text{ROI}}\right)}{\sqrt{\sum_{i=1}^{I}{\left(f_{i}^{\text{Layer}}-\mu^{\text{Layer}}\right)}^{2}}\;\sqrt{\sum_{i=1}^{I}{\left(f_{i}^{\text{ROI}}-\mu^{\text{ROI}}\right)}^{2}}}\;,$$

where, $\mu^{\text{Layer}}$ and $\mu^{\text{ROI}}$ are the means of ${\left\{f_{i}^{\text{Layer}}\right\}}_{i=1}^{I}$ and ${\left\{f_{i}^{\text{ROI}}\right\}}_{i=1}^{I}$, respectively. This measure is based on the correlation calculated with individual units, not just on the peaks of the distributions. Therefore, we took the variability in the distribution into account so that highly fluctuating top ROIs in each layer lead to low BH scores. Randomly selected 1000 units per layer were used in the decoding analysis. The remaining DNN units after excluding poorly predicted ones were used. To match the number of layers used to compute the BH score across DNNs, we computed the score with the first, last, and randomly selected three intermediate layers. The random layer selection and BH score computation were repeated 10,000 times, and the mean score is reported.

A complementary evaluation can be performed with encoding analysis by exchanging fMRI voxels and DNN units in BH score computation (Figure 1B). In the encoding analysis, we identified the DNN layer with the best encoding accuracy for each fMRI voxel (top layer). The encoding-based BH score is defined by the Spearman rank correlation coefficient between the ROI number and the top layer number across fMRI voxels. We mainly report the average of the decoding-based and encoding-based BH scores, while we examine the consistency between these two metrics.

In the current study, we performed the decoding and encoding analyses using fMRI data collected by Shen et al. (2019) (see STAR Methods: “fMRI dataset”). This dataset is composed of fMRI activity of three subjects viewing 1250 natural object images from ImageNet (2011, fall release; Deng et al., 2009). fMRI responses to 1200 and 50 natural object images were separately used as training and test data for the decoders. The top ROIs were computed using fMRI data from individual subjects separately. Thus, the BH score could be computed using the fMRI data from each subject. We report the consistency of BH scores across the three subjects. Unless stated otherwise, we calculated the Spearman rank correlation coefficient by pooling all top ROIs across three subjects.

### Comparison of BH scores across DNNs

**Figure 2:**
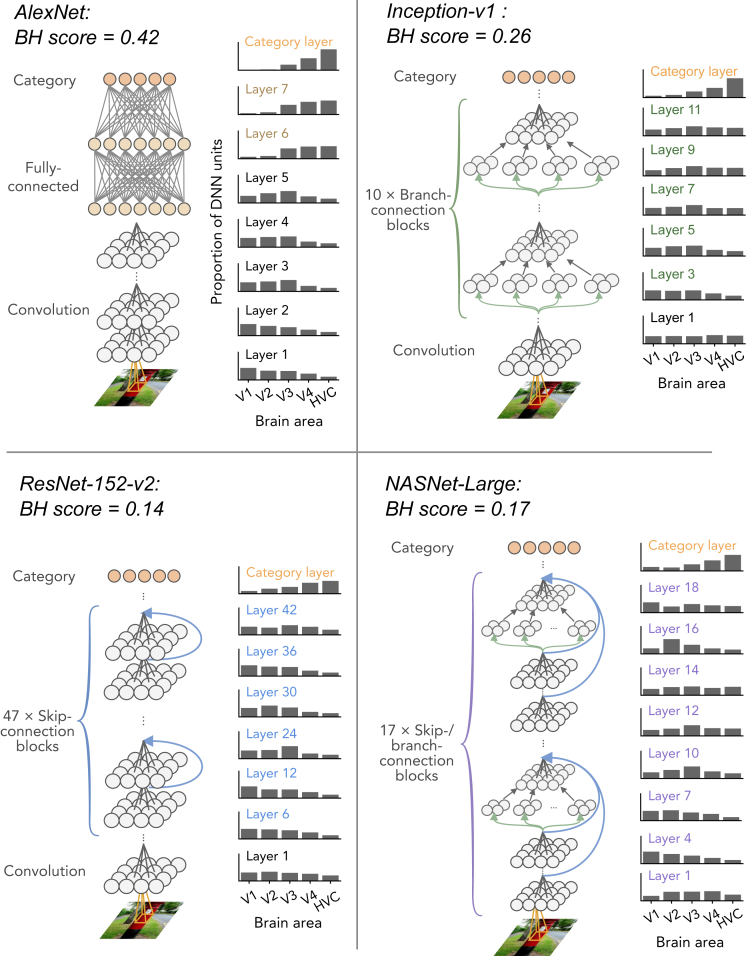
Top ROI distributions and BH scores for representative DNNs The distributions of top ROIs for each layer of AlexNet, Inception-v1, ResNet-152-v2, and NASNet-Large are shown with schematics of their architectures. Histograms are normalized for each layer by the total number of counted DNN units. To match the number of layers used to calculate the BH score across DNNs, BH scores were computed by randomly choosing five DNN layers. The mean BH scores across 10,000 random selections are shown above.

We compared BH scores between 29 representative DNNs (Table 1). All DNNs were pre-trained to classify 1000 object categories on ImageNet (Deng et al., 2009). In the decoding analysis, these DNNs showed different tendencies in their distribution of top ROIs of DNN layers (Figure 2; also see Figure S1). For example, DNNs with FC layers (e.g., AlexNet; Krizhevsky et al., 2012 and the VGG family) (Figure S1) show a clear gradual shift of top ROI distributions from lower to higher visual areas along with the hierarchy of DNN layers. Unit activations in early convolutional layers were better predicted from the lower visual areas, those in late convolutional layers were better predicted from V3, and those in the FC layers were better predicted from V4 or HVC. This gradual hierarchical correspondence between the DNN layers and the visual cortical areas led to high BH scores (e.g., BH score = 0.42 for AlexNet). Inception-v1 (Szegedy et al., 2014), which consists of blocks with branch-connections (inception module), shows flat distributions of top ROIs. Similar flat distributions are also observed in other DNNs with branch-connections (e.g., the Inception family, NASNet [Zoph et al., 2018] and PNASNet [Liu et al., 2018]; Figure S1). Although the top ROI distributions in the category layers show a clear peak at HVC, the flat distributions in the other layers obscure the gradual shift along the hierarchy of DNN layers, resulting in low BH scores (e.g., BH score = 0.27 for Inception-v1). One possible reason for the flattened distributions of top ROIs is that different sizes of convolution and pooling operations are parallely applied in branch-connections, and DNN units corresponding to different brain areas that tend to co-exist within the individual layers. In DNNs with skip-connections (residual module) such as ResNet-152-v2 (He et al., 2015), NASNet-Large (Zoph et al., 2018), and variants of these DNNs (e.g., the DenseNet family and PNASNet-Large [Liu et al., 2018]), the peaks of top ROI distributions non-monotonically swing between V1 and V3. Despite the peak of HVC at the category layers, these swinging peaks in the other layers disrupted the gradual shift of top ROI distribution along the hierarchy and led to low BH scores (e.g., 0.14 for Resnet-152-v2 and 0.17 for NASNet-Large; Figure S1). This tendency may be caused by bypassing of representations via skip-connections: representations similar to lower visual areas are bypassed from lower to higher DNN layers via the skip-connections, and the higher layers tend to include units that are well predicted from fMRI responses in the lower visual areas. In the encoding analysis, the DNNs exhibited consistent tendencies in the distribution of top layers of each brain region (Figure S6).

**Figure 3:**
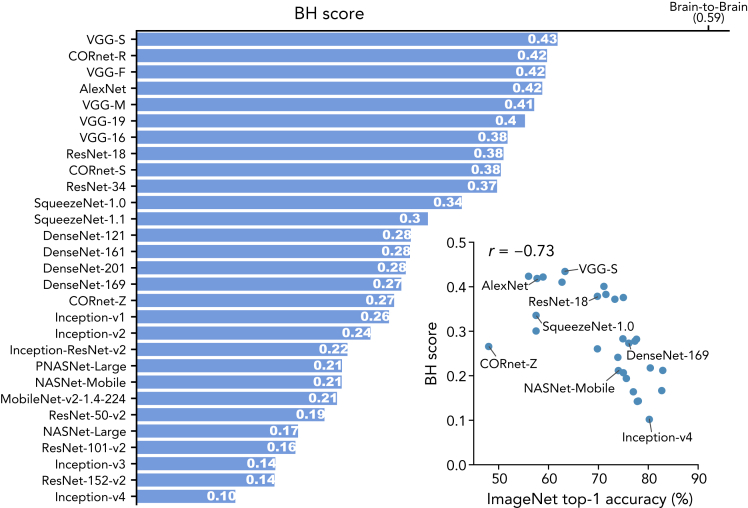
BH scores and ImageNet top-1 accuracies BH scores and ImageNet top-1 accuracies. The BH scores for the 29 DNNs are shown in the descending order of the BH scores. The brain-to-brain score was calculated by predicting fMRI voxel activities from those of other subjects (0.59), which is considered an upper bound (see STAR Methods: “Brain hierarchy score” for the computation procedure). In the right panel, the BH scores for the 29 DNNs are plotted against the ImageNet top-1 accuracy.

By comparing these 29 DNNs, we found that DNNs with simple architecture (e.g., AlexNet, the VGG family, and the CORnet family) exhibited relatively high BH scores (Figure 3). In contrast, DNNs with elaborate architecture and high image recognition performance, such as the DenseNet family, the ResNet family, and the Inception family, showed low BH scores. BH scores were negatively correlated with the ImageNet top-1 accuracies across the DNNs (Figure 3; *r* = −0.73, permutation test, *p* < 0.01), indicating that high-performance DNNs are *not* brain-like if hierarchy is considered.

To examine whether this negative correlation is robust to the measurement of image recognition performance, we evaluated DNNs' image recognition accuracies on image datasets other than ImageNet: Caltech-101 (Fei-Fei et al., 2007) and Caltech-256 (Griffin et al., 2007). A multinomial logistic regression classifier was trained using the second last layer of each DNN as input. The classifiers were trained and tested with the training and test data of each dataset. BH scores were also negatively correlated with image recognition accuracy evaluated on Caltech-101 data and that evaluated on Caltech-256 data (Figure S2).

**Figure 4:**
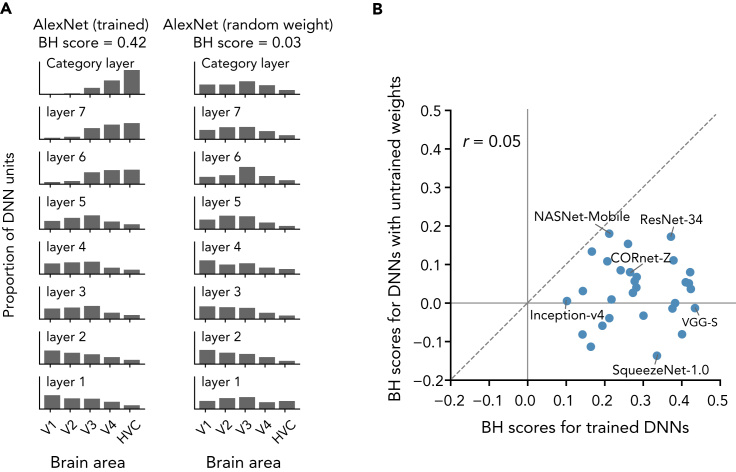
BH scores for trained and untrained DNNs (A) The top ROI distributions and BH scores for the original trained AlexNet and the untrained AlexNet. Both DNNs had the same architecture but the untrained AlexNet had random weights. (B) BH scores for trained and untrained DNNs. The BH scores for the original trained 29 DNNs and for the untrained DNNs are shown in a scatter plot.

To examine whether training is necessary for DNNs to yield brain-like hierarchical representations, we compared the degree of hierarchical homology between trained and untrained (i.e., with random weights) versions of the 29 DNNs. To construct untrained DNNs, we used randomly initialized weights provided in the original implementation of the DNNs. Overall, the untrained DNNs exhibited lower BH scores than DNNs trained on ImageNet (Figure 4). The monotonic shift of the distribution of the top ROIs along the hierarchy of DNN layers was deteriorated by replacing DNN's trained weights with random values (Figure 4A). The unit activations in the untrained DNNs were less predictable, particularly in higher layers (e.g., layers 6 and 7, and the category layer in AlexNet), from the fMRI responses in higher visual areas (V4 and HVC) compared with those in the trained DNNs. This effect resulted in flattened, middle ROI-peaked, or lower ROI-peaked distributions of the top ROIs in each layer, and degraded the BH scores of DNNs with random weights. Similar degradation of BH scores was observed in the majority of tested DNNs (Figure 4B). The results indicate that hierarchical homology of DNNs to the brain does not arise only from their architectures, but also requires training with image data.

### Robustness of the BH score

We defined the BH score based on several optional choices of procedures. Here we consider the robustness of the BH score to some of these choices. In the calculation of the BH score, we excluded DNN units whose decoding accuracies did not exceed a predefined threshold for any ROIs (student's *t*-test, *p* < 0.05). On average, 42.9% of DNN units were excluded by this procedure. We computed BH scores without this unit exclusion and compared them with the original BH scores (Figure S3A). BH scores with and without unit selection were highly correlated across the 29 DNNs (*r* = 0.91). In addition, we found highly correlated scores when raw unit activations were used instead of absolute values for the first layer (Figure S3B, *r* = 0.98). Although the scores with and without these procedures were strongly correlated, the original BH scores tended to take a wide range of values, indicating that these procedures are effective in detecting monotonic shifts with more sensitivity.

We also examined the consistency of BH scores across fMRI data from individual subjects. We computed BH scores using fMRI data for each subject, and found that similar scores were observed across subjects (Figure S4). The results suggest that the BH score does not depend on the particular brain used for calculation.

We examined whether similar comparison results were consistently observed with each of the decoding and encoding analyses. Here, we computed the BH score based on either decoding or encoding analysis, and those two measures were compared (Figures S5 and S6). The decoding-based BH scores were positively correlated with the encoding-based BH scores (Figure S5; *r* = 0.54). Both scores were also negatively correlated to ImageNet top-1 accuracies (*r* = −0.72 [decoding] and *r* = −0.47 [encoding]). Although these measures do not perfectly agree, the overall tendency is similar and the relation to the ImageNet top-1 accuracies appears to be robust regardless of the choice of the analysis method.

**Figure 5:**
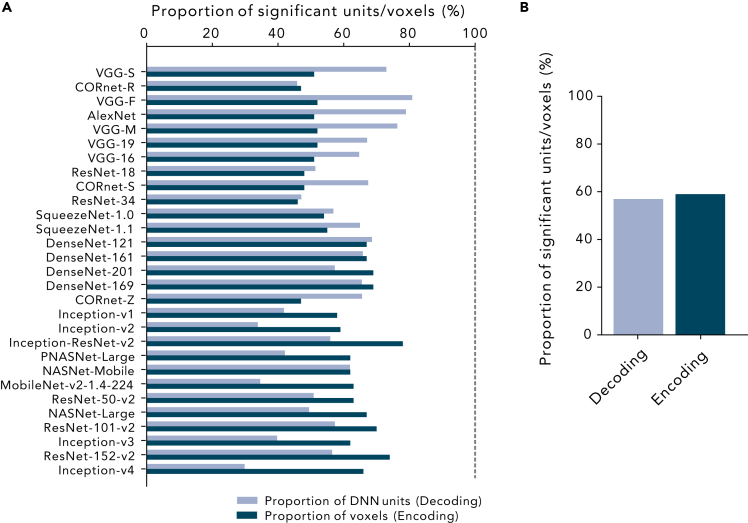
Proportion of significantly predicted DNN units and voxels (A) Proportion of significantly predicted DNN units in the decoding analysis (light blue) and significantly predicted voxels in the encoding analysis (dark blue). The results for each DNN are shown. DNNs are sorted by their BH scores. (B) The average proportion of significantly predicted units/voxels across 29 DNNs.

To further characterize the difference between the encoding and decoding approaches, we performed post-hoc analyses to see what proportion of fMRI voxels and DNN units effectively contribute to the decoding- and encoding-based BH scores. First, we obtained the proportion of significantly predicted DNN units and fMRI voxels that were included in the calculation of decoding-based and encoding-based BH scores, respectively (see STAR Methods: “Decoding analysis” and “Encoding analysis”; Figure 5). In the decoding analysis, 56.93% of DNN units on average showed significantly higher than chance-level prediction accuracy. In the encoding analysis, a similar proportion of the units (58.97%) showed significantly higher than chance-level prediction accuracy.

**Figure 6:**
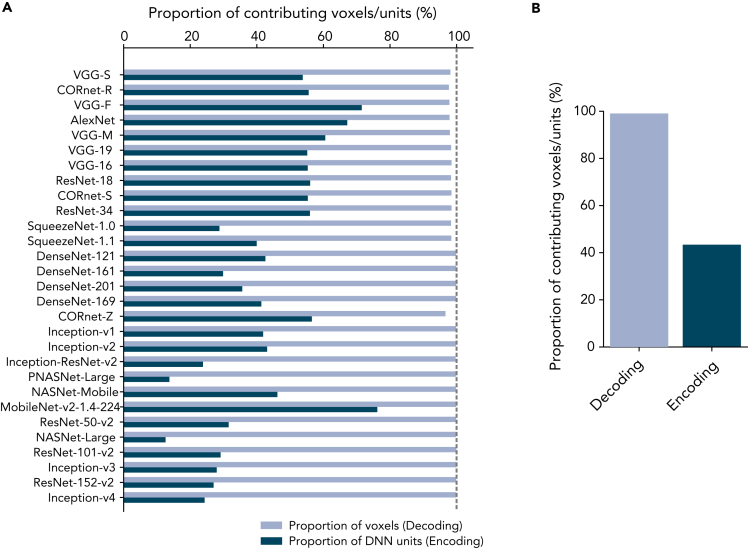
Proportion of input voxels and DNN units contributing for the prediction (A) Proportion of voxels/units that contribute for the prediction accuracy in the decoding/encoding analysis. The results for each DNN are shown. The DNNs are sorted by their BH scores. (B) The average of the proportion of contributing voxels/units across DNNs.

Next, we examined the proportion of input features, fMRI voxels in decoding and DNN units in encoding, that were selected for prediction of at least one of their target variables (see STAR Methods: “Decoding analysis” and “Encoding analysis”). In our main analyses, we selected 500 voxels/units that were most correlated with each target variable (see STAR Methods: “Feature importance analysis”). In the decoding analysis, almost all the voxels were selected in at least one of the predictions regardless of target DNNs. In contrast, a limited portion of DNN units were selected from at least one of the predictions in the encoding analysis. Although the proportion of selected units varied across DNNs, overall a majority of DNN units were not selected for any of the predictions of voxel activations (Figure 6). Qualitatively similar results were observed with different selection thresholds and criteria.

The encoding-based BH scores showed a weaker negative correlation with ImageNet top-1 accuracies than the decoding-based scores. A post-hoc analysis revealed a lower correlation between subjects in the encoding-based BH scores than in the decoding-based BH scores (Figure S4). This lesser consistency across subjects might reduce the correlation between the encoding-based BH score and ImageNet top-1 accuracy.

We have used the BH scores calculated with sets of randomly selected 5 DNN layers to match the number of layers across DNNs. We examined whether similar results could be obtained even when we excluded this procedure. We computed BH scores without layer selection and compared them with our original BH scores. The rank correlation between those two types of BH scores across the 29 DNNs was 0.91, indicating that the BH score ranking was not much affected by layer selection.

We examined whether the BH score depends on our choice of ROIs. Specifically, our definition of HVC includes the large portion of the ventral visual cortex, summarizing the finer architecture and potentially underestimating the hierarchical correspondence to DNN layers. Thus, we tested whether dividing HVC affects the BH score. Because it is difficult to define a hierarchical order between the subregions delineated by the conventional functional anatomy, we employed a data-driven approach: we divided HVC into three regions based on the principal gradient defined by functional connectivity of resting-state activity (Margulies et al., 2016; see STAR Methods: “Region of interest”) and calculated BH scores with 7 ROIs (e.g., V1, V2, V3, V4, HVC-1, HVC-2, and HVC-3). The BH scores with 7 ROIs were highly correlated to those with 5 ROIs across the 29 DNNs (Figure S7A, *r* = 0.98), and negatively correlated to ImageNet top-1 accuracy (Figure S7B, *r* = −0.73). Although the top ROI distributions at the category layers tended to peak at HVC with 5 ROIs, they peaked at HVC-1 with 7 ROIs, not at HVC-2 or -3 (Figure S8). This suggests that the category layers in the DNNs we tested have similar representations to the lower portion of HVC, and that the subdivision of HVC does not improve the hierarchical correspondence to the DNNs.

We also applied the principal gradient as a measure of data-driven parcellation of the entire ventral visual cortex we used in this study. We divided the visual cortex into 5 or 10 ROIs based on Gradient1 of the principal gradient (see STAR Methods: “Region of interest”) and calculated BH scores with the 5 or 10 ROIs. However, the BH scores had substantially small values regardless of the number of ROIs (*r* = 0.04 ± 0.04 for 5 ROIs and *r* = 0.02 ± 0.06 for 10 ROIs; averaged over 29 DNNs). The result suggests that the principal gradient does not necessarily allow us to investigate a detailed hierarchical correspondence in the visual cortex whereas it is effective in capturing a global hierarchy of the entire cortex.

### Measurement modality of brain activity

**Figure 7:**
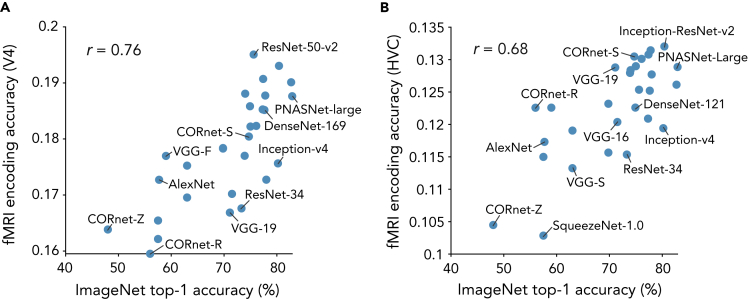
fMRI encoding accuracy and ImageNet top-1 accuracy (A) fMRI V4 encoding accuracy and ImageNet top-1 accuracy. Responses of individual voxels in V4 were predicted from unit activations of each DNN. The mean prediction accuracies (encoding accuracies) across the voxels are plotted against the ImageNet top-1 accuracies. (B) fMRI higher visual cortex (HVC) encoding accuracy and ImageNet top-1 accuracy. The mean encoding accuracies for HVC are plotted against the ImageNet top-1 accuracies.

The Brain Score study (Schrimpf et al., 2018) and our current study employed brain data obtained by different measurement methods: electrophysiological recordings from monkeys and fMRI from humans. To examine whether the measurement modality is critical to the evaluation of brain-DNN similarity, we performed an encoding analysis similar to the previous studies (Schrimpf et al., 2018; Yamins et al., 2014; Yamins and DiCarlo, 2016) on our fMRI data. In accordance with the brain regions examined in the previous studies, we selected V4 and HVC for this encoding analysis. The responses of individual voxels in V4 and HVC were predicted from the activations of DNN units in each DNN (Figure 7; STAR Methods: “Encoding analysis”). Consistent with the previous finding, we observed a positive correlation between ImageNet top-1 accuracy and encoding accuracy across DNNs (*r* = 0.76 for V4; *r* = 0.68 for HVC). The results suggest that electrophysiology and fMRI provide a similar evaluation of brain-DNN similarity in individual areas.

### Similarity between DNNs and single brain regions

Recent studies have used representational similarity analysis to evaluate the similarity between a DNN and human IT using fMRI, reporting a negative correlation between the representational similarity to human IT and ImageNet top-1 accuracy across DNNs (*r* = −0.47 in Jozwik et al. (2019) and *r* = −0.38 in Storrs et al. (2020)). We replicated the results using fMRI data in the current study (*r* = −0.57; Figure S9). Although these results are based on individual brain areas, not hierarchy, the lower similarity of high-performance DNNs to the higher visual areas (human IT or HVC) might contribute to poor hierarchical correspondence, accounting for the negative correlation between BH scores and image recognition performance. However, it remains unclear why representational similarity and predictive (encoding or decoding) accuracy exhibited opposite correlations to image recognition performance when measured in individual areas.

### BH scores and DNN architectural components

What components of architecture are critical for brain-like hierarchical representations in DNNs? We examined how BH scores are explained by five representative components of DNN architectures: the presence of FC layers, the presence of skip-connections, the presence of branch-connections, the total number of convolutional and FC layers (i.e., depth), and the number of weight parameters. Note that to define the depth of a DNN, we counted not only the representative layers but also those not used in the computation of the BH score. For the first three components, we compared the mean BH score between the presence and the absence of each component. Correlations with the BH score were calculated for the depth and weight parameters. Because only two DNNs (CORnet-R and CORnet-S) had recurrent connections among the DNNs we tested in the current study, the difference owing to the presence of recurrent connections was not quantitatively examined (BH scores for CORnet-R and CORnet-S are 0.44 and 0.41, which is relatively high among the tested DNNs).

**Figure 8:**
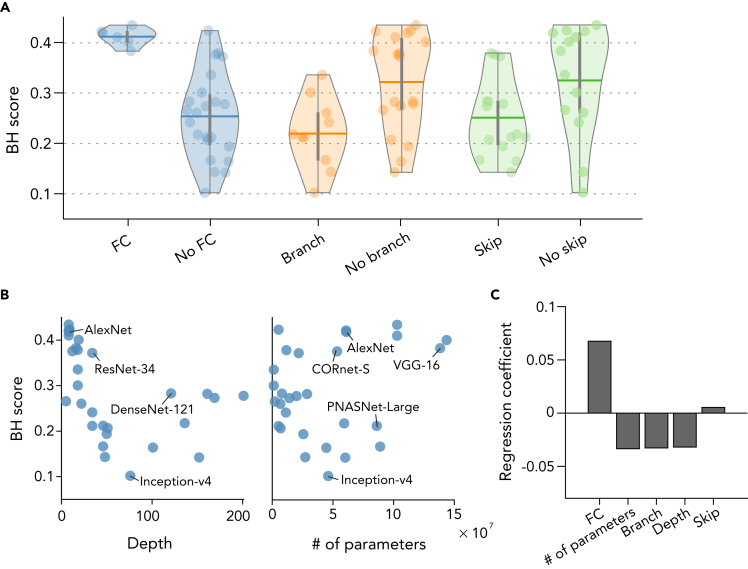
Relationship between BH scores and DNN architectures (A) Comparison between DNNs with and without fully-connected (FC) layers, skip-connections and branch-connections. Thick horizontal lines show the mean BH score across the DNNs. Each dot denotes an individual DNN. Vertical gray lines indicate the interquartile ranges. Shaded areas are the full ranges of data points. (B) Relationship to DNN depth and the number of weight parameters. Each dot denotes individual DNNs. (C) Regression analysis. A linear regression model was fitted to explain the BH score with five architectural components. The resultant standardized regression coefficients are shown for each component.

DNNs with FC layers exhibited markedly higher BH scores than those without FC layers (*d’* = 3.07 [*d*-prime: the mean difference normalized by the standard deviation]; Figure 8A), whereas those with skip-connections and branch-connections showed moderately lower BH scores: *d’* = −0.78 for skip-connections and *d’* = −1.23 for branch-connections (Figure 8A). We also found a negative correlation between BH scores and depth (*r* = −0.47; Figure 8B), and a weak positive correlation between BH scores and the number of weight parameters (*r* = 0.26; Figure 8B). Complementary regression analysis, in which BH scores were modeled as a linear weighted sum of variables parameterizing the five components, also indicated a greater contribution of the presence of FC layers compared with the other components (Figure 8C).

**Figure 9:**
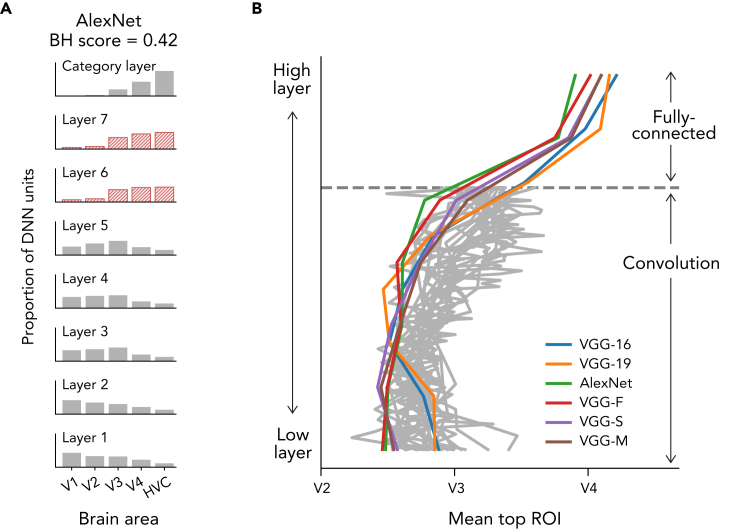
FC layers and the shift of top ROIs (A) The distributions of top ROIs for AlexNet. The distributions for the FC layers are shown in red. (B) The mean top ROI across all layers for the 29 DNNs. For each DNN, we computed the mean of the distribution of each layer except for the last layer, and plotted it as a function of the depth of the DNN layer. The DNNs with FC layers are colored, while the DNNs without FC layers are shown in grey. The depth of the DNN layer has been rescaled for visualization purposes.

How do FC layers contribute to the high degree of hierarchical homology? As an example, in AlexNet, the distributions of top ROIs for FC layers have peaks at HVC (Figure 9A), leading to a large shift in distribution. To examine whether this tendency is consistently observed for other DNNs with FC layers, we computed the mean top ROI number of each layer and plotted it as a function of the layer number for the 29 DNNs (Figure 9B). Whereas the mean top ROI numbers in the higher layers shifted up to higher visual areas in the DNNs with FC layers, the shift stopped at mid-level visual areas in DNNs without FC layers.

### Experimental manipulation of DNN architecture

To complement the observations from pre-trained DNNs described above, we experimentally manipulated DNN architectures in a base DNN, AlexNet consisting of five convolutional layers, two fully-connected (FC) layers, and one category layer without skip- or branch-connections. We created these variants of the base DNN and compared their BH scores. These DNNs were trained on the same ImageNet dataset.

**Figure 10:**
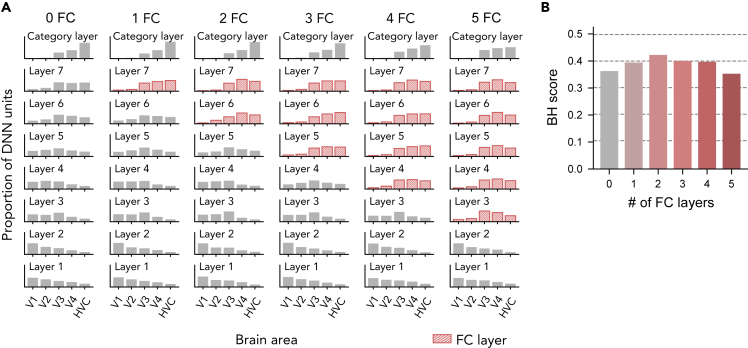
Effect of the number of FC layers (A) Eight-layer DNNs with different numbers of FC layers were trained on ImageNet (see STAR Methods: “Manipulation of DNN architecture” for the details of the architectures). Their top ROI distributions are shown. FC layers are indicated in red. (B) BH scores for the different numbers of FC layers.

First, we manipulated the number of FC layers from zero to five while maintaining the other architectural characteristics (STAR Methods: “Manipulation of DNN architecture”). The DNNs with 0, 1, 2, 3, 4, and 5 FC layers achieved 0.55, 0.57, 0.56, 0.51, and 0.46 ImageNet top-1 accuracies, respectively. The DNN with no FC layers showed a gradual shift of the top ROI distribution up to the second last layer, but had a relatively large gap between the second last layer and the last layer (Figure 10A). In contrast, in DNNs with one, two, or three FC layers, the top ROI distribution gradually shifted over the layers. With even more FC layers, the shift of top ROIs over the convolutional layers became less gradual, and the last convolutional layer and the first FC layer exhibited a large gap. Thus, the BH score was highest at the DNNs with two FC layers (Figure 10B).

The effect of FC layers on the BH score was also tested for DNNs with different base architectures (i.e., ResNet-based and Inception-based DNNs). The DNNs with two FC layers also had higher BH scores than DNNs with no FC layers for both ResNet-based DNNs (0.41 for two FC layers and 0.38 for zero FC layers) and Inception-based DNNs (0.41 for two FC layers and 0.39 for zero FC layers). In our comparison of the 29 pre-trained DNNs, all of the DNNs with FC layers (i.e., AlexNet and the VGG family) had two FC layers. This moderate number of FC layers is likely to have produced high BH scores.

To examine the effect of the number of FC layers in terms of the similarity to single brain regions, we also compared encoding accuracy for each brain area across the DNNs with different numbers of FC layers (see “Similarity between DNNs and single brain regions” for methods). Although the DNN with 2 FC layers showed the highest BH score (Figure 10B), the DNNs with the best encoding accuracy differed depending on the predicted brain area: higher brain areas tended to be better explained by the DNNs with larger numbers of FC layers (Figure S10). This result indicates a dissociation between hierarchical homology captured by the BH score and the similarity to single brain regions.

Convolutional layers and FC layers differ in two main aspects: kernel size and the number of channels. While each unit in an FC layer has connections from all units in the previous layer, units in convolutional layers have spatially limited connections. The spatial range of connections allowed in a convolutional layer is specified by the kernel size. In addition, the number of channels is generally different between convolutional layers and FC layers. The number of channels in an FC layer is typically set to several thousand, whereas that in convolutional layers is set to several hundred. To examine which component is critical for explaining high BH scores of DNNs with FC layers, we tested how each component in FC layers affected the BH score.

**Figure 11:**
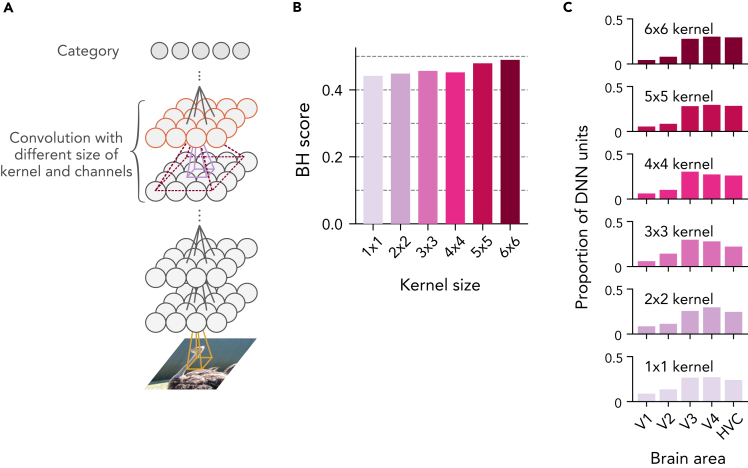
Effects of the width of spatial integration, and the number of channels (A) We used a seven-layer DNN while changing the kernel size at the second last layer (layer 6) indicated in red (see STAR Methods: “Deep neural networks”). (B) BH scores with varied kernel sizes. (C) Top ROI distributions at layer 6 with varied kernel sizes.

To examine the effects of kernel size, we constructed a DNN with six convolutional layers and one category (fully-connected) layer, and manipulated the kernel size of the last convolutional layer from one to six (Figure 11A; see STAR Methods: “Manipulation of DNN architecture”). When the kernel size of the DNN is six, each unit in the last convolutional layer has connections from all units in the second last layer. In other words, the last convolutional layer of this DNN is equivalent to an FC layer. By changing this kernel size systematically, we examined how the BH score and the representation of this layer changed depending on this parameter. As the kernel size became larger, the BH score increased (Figure 11B) and the distribution of top ROIs for the last convolutional layer was centered at higher visual areas with some fluctuations (Figure 11C).

We also tested how other architectural characteristics (i.e., presence of skip-connections, presence of branch-connections, and depth) affect the BH score by manipulating either of them in base DNNs (AlexNet for the presence of skip- or branch-connections and VGG-16 for depth; STAR Methods: “Manipulation of DNN architecture”). Skip- and branch-connections were introduced by replacing convolutional layers with residual blocks of ResNet-18 and inception modules of Inception-v1, respectively. The depth was manipulated by inserting additional convolutional layers. Note that the additional convolutional layers were not included in the calculation of BH scores. Because the number of weight parameters could not be changed independently, it was not considered here.

**Figure 12:**
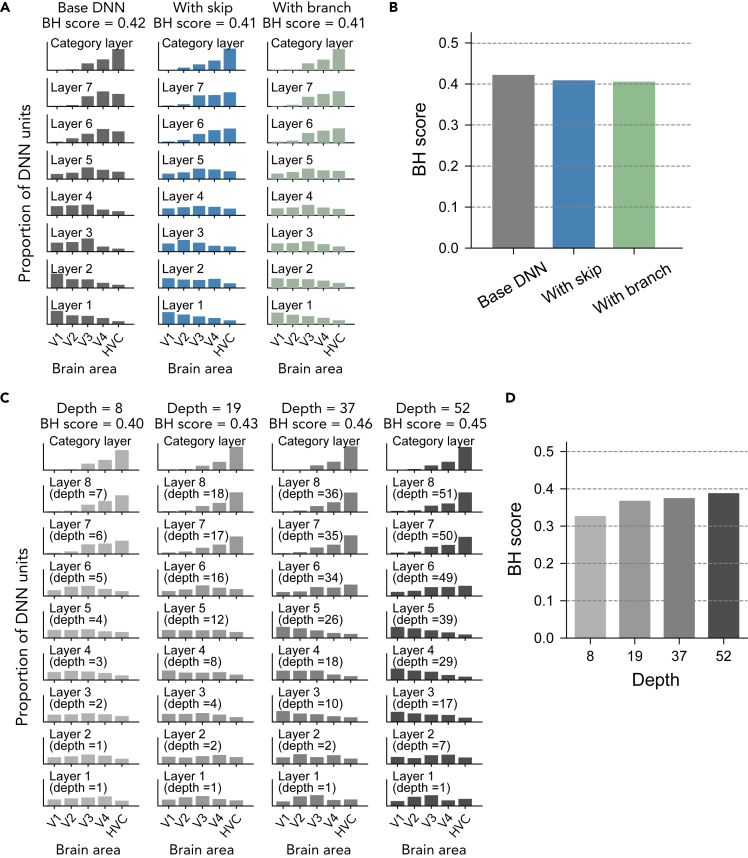
Effects of depth, skip-connections, and branch-connections (A) Effect of skip-connections and branch-connections. A DNN with pure convolutional layers, a DNN with skip-connections and a DNN with branch-connections were prepared and trained on ImageNet using the same procedure (see STAR Methods: “Deep neural network training” for details of the architectures). (B) BH scores for the base DNN, DNN with skip-connections and DNN with branch-connections. (C) Effect of depth. Four DNNs with different depths were prepared by inserting convolution layers into each layer. The layer number and depth at each layer are also shown. (D) BH scores for DNNs with different depths.

Consistent with the tendency among the 29 pre-trained DNNs, the distributions of top ROIs for DNNs with skip-connections and branch-connections were relatively flat compared with the base DNN. In addition, the top ROI distributions for the DNN with skip-connections did not monotonically shift from lower to higher visual areas: the centroid of the distribution shifted to higher visual areas at layer 2, then back to lower areas at layer 3 (Figure 12A). As a result, those DNNs showed slightly lower BH scores than the base DNN without them (Figure 12B). When the depth was manipulated, BH scores of deeper DNNs tended to be higher with sharper peaks (Figures 12C and 12D).

## Discussion

In the current study, we presented a method for quantifying the hierarchical similarity between the human brain and deep neural networks (DNNs) and its applications in an attempt to elucidate the characteristics that make DNNs hierarchically brain-like. We characterized individual DNN units by their best decodable visual areas (top ROIs) in fMRI decoding analysis and quantified the correspondence of hierarchical representations between the brain and DNNs. The distributions of top ROIs revealed differences in the hierarchical transformation of representations between DNNs (Figure 2). We also performed similar characterization by exchanging the DNN units and fMRI voxels (i.e., encoding analysis). By combining the results of the decoding and encoding analyses, our proposed metric, i.e.,the brain hierarchy (BH) score, was negatively correlated with image recognition performance across DNNs (Figure 3), suggesting that high-performance DNNs are not necessarily brain-like. This negative correlation with image recognition performance was consistently observed with different image datasets (Figure S2). The omission of training decreased BH scores of the DNNs (Figure 4), indicating the importance of DNN training for hierarchical homology with the human brain. The BH score was robust to optional choices about the unit selection and the processing of unit activations in the first layer (Figure S3). Complementary encoding analysis also provided similar comparison results across DNNs (Figures S5 and S6). By comparing DNNs with different architectures, we identified architectural characteristics that were associated with the degree of hierarchical homology (Figure 8). DNNs with fully-connected (FC) layers exhibited higher BH scores, and DNNs with skip- and branch-connections exhibited lower BH scores by smaller margins. DNNs with FC layers gradually developed internal representations from those similar to lower visual areas to those similar to higher visual areas, whereas DNNs without FC layers lacked layers whose representations were similar to higher visual areas (Figure 9). This observation was also confirmed by a manipulation experiment (Figure 10). Additional experiments provided further support for the importance of broad spatial integration at FC layers (Figure 11). The presence of skip-connections and branch-connections tended to degrade the degree of hierarchical homology by flattening and non-monotonically swinging the top ROI distributions, respectively (Figure 12).

The BH score is based on both the decoding of individual DNN units and the encoding of individual voxels. BH scores based solely on either decoding or encoding showed similar results (Figure S5), suggesting the robustness of the results to be the choice of the analysis method. Many previous studies rely on the encoding analysis to assess the similarity between the representation of the brain and DNNs (e.g., Schrimpf et al., 2018; Yamins et al., 2014; Yamins and DiCarlo, 2016; Zhuang et al., 2017; Kell et al., 2018; Cadena et al., 2019). Encoding models can provide descriptions of how neural responses emerged from features in the external world and allow us to directly compare different computational models of the brain (Naselaris et al., 2011; Kriegeskorte, 2011). In addition, encoding approaches can exploit higher order interaction between DNN features. Representational similarity analysis has also been used to detect the similarities to the brain based on the activation patterns across those units (Jozwik et al., 2019; Khaligh-Razavi and Kriegeskorte, 2014; Storrs et al., 2020). Most of these studies applied dimensionality reduction techniques to the DNN activation patterns because of their high dimensionality, thus potentially overlooking fine representations encoded in individual units (Figures 5 and 6).

Although encoding approaches have methodological benefits as described above, our results indicated potential advantages of the decoding analysis in relating DNNs and the brain. First, the portion of input variables (DNN units in encoding and fMRI voxels in decoding) used for at least one prediction is markedly smaller in encoding than in decoding (Figure 6). This indicates a possibility that only a small subset of DNN units could account for brain activity, and thus, the encoding approach may only provide a partial characterization of an entire DNN. Second, the comparison of the inter-subject consistency showed that the decoding-based BH score tended to be more consistent across subjects (Figure S4), which would increase its sensitivity to detect the brain–DNN homology. Note that these observations were found in post-hoc analyses, and require further confirmation with independent datasets.

Our comparative results using the BH score were robust to several choices of procedures. The original BH score was strongly correlated with those computed without the unit exclusion procedure (Figure S3A) or the nonlinear transformation (Figure S3B) across the 29 DNNs. Meanwhile, the raw values of the BH score were systematically affected by these procedures. The unit exclusion procedure tended to increase/decrease the BH score for DNNs with high/low scores, respectively. These procedures generally broadened the range of BH scores, suggesting that they may improve the sensitivity of the BH score.

The BH score was also highly consistent across fMRI datasets from different subjects (Figure S4), consistent with our previous study showing high correlations of the decoding accuracies of individual DNN units between different subjects (Horikawa and Kamitani, 2017). Although the BH score showed high consistency across normal subjects, brain disorders such as schizophrenia have recently been proposed to be associated with disorganized object representations in the brain (Nishida et al., 2020). The BH score based on fMRI responses from participants with such disorders may reveal functional differences compared with typical brains.

In the current study, we found that the BH score was negatively correlated with image recognition performance across the 29 pre-trained DNNs (Figure 3). Thus, high-performance DNNs do not necessarily exhibit hierarchical representations that are similar to the brain. Although early work suggested that DNNs with improved recognition performance are likely to provide better computational models of the brain (Yamins and DiCarlo, 2016), a recent study (Schrimpf et al., 2018) reported that image recognition performance and brain–DNN similarity was more weakly correlated in recently developed high-performance DNNs (DNNs with ≥70% ImageNet top-1 accuracy). Our comparative results using the BH score also showed a dissociation between image recognition performance and similarity to the brain. As one of the gaps in the object recognition process between DNNs and the brain, a recent computer vision study suggested that DNNs trained on ImageNet tended to classify object images according to their textures, whereas humans classified object images based on their shapes (Geirhos et al., 2019). A subsequent study quantitatively compared this texture bias between AlexNet and ResNet-50, and showed that the global-pooling operation in ResNet-50 largely removes shape information and strengthens the texture bias (Hermann and Kornblith, 2019). Although both DNNs have a strong texture bias, AlexNet preserves relatively richer shape information in their FC layers. This suggests that FC layers mitigate DNNs' texture bias and make processing in the higher layers more brain-like, which may explain our comparative results between DNNs with and without FC layers. In contrast, because FC layers have large number of weight parameters and increase the risk of overfitting, global-pooling operations are more commonly adopted in recent high-performance DNNs (Szegedy et al., 2014; He et al., 2015; Zoph et al., 2018; Liu et al., 2018). Likewise, elucidating the differences between high-performance DNNs and the brain remains an important challenge, which, if solved, would provide important insights into the properties of DNNs.

In addition to the quantification of the brain–DNN hierarchical homology with the BH score, we characterized individual DNN layers by the distributions of their top ROIs. The distributions of top ROIs tended to show specific patterns depending on the architectures of DNNs. DNNs with FC layers (e.g., AlexNet and the VGG family) showed unimodal and sharp distributions at the layer. The peak of the distribution was monotonically shifted from lower to higher visual areas along with the hierarchy of the DNN layers, leading to high BH scores for those DNNs. In contrast, DNNs with branch-connections (e.g., the Inception family, NASNet, and PNASNet) tended to have flat distributions. Because the branch-connections develop their features through parallel convolutions with different kernel sizes, representations corresponding to different visual areas may be mixed into single concatenated layers, flattening the distributions. In the DNNs with skip-connections (e.g., the ResNet family, the DenseNet family, NASNet, and PNASNet), the peak of the distribution tended to oscillate between V1 and V3, possibly reflecting the bypassing of representations between lower and higher layers. Although brain regions are connected not only by sequential single feedforward path but also by branch-like or skip-like connections (Felleman and Van Essen, 1991; Sporns and Zwi, 2004), DNNs with such connections do not yield hierarchical representations similar to the brain. Instead, DNNs consisting of single-path sequential feedforward connections acquire more hierarchically similar representations to the brain, suggesting that such plain feedforward connections among brain regions play a dominant role in forming hierarchical representations in the brain.

Although deeper DNNs tended to have lower BH scores among the 29 pre-trained DNNs (Figure 8B), the opposite tendency was observed in the DNNs trained after their depth was manipulated (Figures 12C and 12D). Many of the deeper pre-trained DNNs had skip-connections in their architectures. Skip-connections are often used to mitigate the risk of gradient vanishing in very deep networks (He et al., 2015). The covariation of these architectural factors in the 29 pre-trained DNNs may account for the discrepancy in the effect of depth found in the manipulation experiment.

Our results suggest that full connections in the last few layers (FC layers) make the representations similar to those in the higher visual areas and thus lead to greater hierarchical homology (Figure 10). FC layers can spatially integrate visual features to achieve translation-invariant representation of object categories. fMRI activity in the higher visual areas, including lateral occipital complex (LOC), the fusiform face area (FFA), and the parahippocampal place area (PPA), is associated with processing translation-invariant information of object categories (Carlson et al., 2011). Thus, spatial integration of local visual features may play a common crucial role in hierarchical transformation of spatially invariant visual representation in both DNNs and the brain. In addition, a recent study reported that DNNs with FC layers exhibit better generalizability across datasets than all convolutional DNNs (i.e., DNNs without FC layers) (Zhang et al., 2018). FC layers may also play a critical role in achieving human-level generalizability. In contrast, all convolutional DNNs tend to show better ImageNet top-1 accuracies than DNNs with FC layers. This is presumably because all convolutional DNNs have smaller number of weight parameters owing to weight sharing, thus allowing for efficient learning with a limited amount of training data. Thus, only performance-optimization for a specific task may not lead to brain-like DNNs. Consistent with this view, in the manipulation experiment in which the number of FC layers was changed, the DNN with one FC layer achieved the highest ImageNet top-1 accuracy whereas the DNN with two FC layers showed the highest BH score. This also indicates that a greater degree of hierarchical homology is not necessarily associated with higher object recognition performance.

We found that DNNs with random weights (i.e., untrained DNNs) showed markedly lower BH scores than DNNs trained for the classification task on ImageNet (Figure 4). Similarly, DNNs trained for object classification have shown to have a more similar representation to human IT than untrained DNNs (Storrs et al., 2020). These results suggest that goal-driven task optimization of DNNs is critical for brain-like representations. Another recent study reported that DNNs trained in an unsupervised manner could explain neuronal responses in monkey V1, V4, and IT cortex to a comparable degree to DNNs trained for object classification (Zhuang et al., 2020). Thus, DNNs could acquire brain-like hierarchical homology not only by explicit tasks but also by implicit learning of task-related features.

Although we quantitatively characterized hierarchical homology of visual representations between DNNs and the human brain, the BH score could be used for examining hierarchical homology of representations in different modalities (e.g., auditory representations, tactile representations) or that between DNNs and the brain of other species. Previous studies examined hierarchical homology between a DNN trained for sound classification and the human auditory cortex (Kell et al., 2018) and the hierarchical homology between VGG16 and the mouse visual cortex (Cadena et al., 2019). Another study proposed several types of biologically-feasible DNN that imitate hierarchical representations of the rodent tactile system (Zhuang et al., 2017). It remains unclear which type of DNN best captures the properties of real rodents. The BH score would provide a quantitative tool for comparison of different types of DNN in terms of the similarity to given hierarchical representations in those modalities and species.

### Limitations of the study

The BH score is only applicable to single-path sequential hierarchies of deep neural networks (DNNs) and brains. For the formalization of hierarchy, we assumed that the hierarchy is represented by an ordinal scale (i.e., layer and ROI numbers). Thus, the BH score does not incorporate multi-path or non-sequential hierarchical structures such as branching, collateral (skip) path, or loop (recurrence). Although some DNNs tested in this study have branch-connection, skip-connection, or recurrent-connection, we selected the output layers of the branch-block, skip-block, or recurrent-block (submodules) instead of respective individual layers in the block as multiple layers. Thus, the hierarchy of DNNs was summarized as a single feedforward pathway. In the current study, we focused on DNNs developed for image recognition and the brain regions underlying object recognition (i.e., the ventral visual pathway), in which neural representations are assumed to develop through a single pathway. To quantify the homology between more complex DNNs (e.g., multi-path, and/or recurrent neural networks) and the whole brain network, a more sophisticated metric will be required.

The choice of brain regions (ROIs) and their hierarchical order are critical for the BH score: different sets and orders of ROIs produce different scores in the same DNN. In this study, we selected brain regions in the ventral visual pathway (V1, V2, V3, V4, and higher visual cortex [HVC]), which underlies object recognition. The vital role in object recognition and the structural and functional hierarchy of these areas have been well established in neuroscience research, supporting the notion that our set and ordering of ROIs captured the hierarchy of object recognition. Nevertheless, this ROI selection method based on known functional anatomy is inevitably user-dependent and has the potential to scatter BH scores. Moreover, the prior knowledge-based selection of ROIs has a potential inherent risk of overlooking hierarchy in the brain that is not incorporated in our prior knowledge. Instead of such prior knowledge-based ROIs, brain hierarchy characterized by data-driven approaches (e.g., Margulies et al., 2016) can be used for the assessment of BH scores and yield alternative measures.

As architectural characteristics of interest, we did not focus on the presence of recurrent connections because only one DNN has recurrent connections among the 29 DNNs examined in the current study (Table 1). We limited the DNNs tested here to those trained on the same ImageNet classification task for a fair comparison, and there were few available pre-trained DNNs with recurrent connections satisfying this limitation. Several studies recently developed DNNs with recurrent connections, suggesting that those DNNs capture the dynamics of neuronal responses in the ventral visual areas (Nayebi et al., 2018; Spoerer et al., 2017). Testing new DNNs designed to imitate the processing in the cortex will be helpful for elucidating the importance of recurrent connections in the human visual system.

## STAR★Methods

### Key resources table

**Table:** 

REAGENT or RESOURCE	SOURCE	IDENTIFIER
**Deposited data**		

Unit activation of DNNs	This paper	https://doi.org/10.6084/m9.figshare.12401168
Code for calculating the BH score	This paper	https://github.com/KamitaniLab/BHscore

**Software and algorithms**		

Caffe	Jia et al., 2014	http://caffe.berkeleyvision.org
TensorFlow	Abadi et al., 2016	https://www.tensorflow.org/; RRID: SCR_016345
PyTorch	Paszke et al., 2019	https://pytorch.org/; RRID: SCR_018536

**Other**		

fMRI dataset (raw)	Shen et al., 2019	https://openneuro.org/datasets/ds001506
fMRI dataset (preprocessed)	Shen et al., 2019	https://doi.org/10.6084/m9.figshare.7033577
ImageNet (2011 fall release)	Deng et al., 2009	https://image-net.org/challenges/LSVRC/2011/index.php
Caltech 101	Fei-Fei et al., 2004	http://www.vision.caltech.edu/Image_Datasets/Caltech101/
Caltech 256	Griffin et al., 2007	http://www.vision.caltech.edu/Image_Datasets/Caltech256/

### Resource availability

#### Lead contact

Further information and requests for resources should be directed to and will be fulfilled by the lead contact, Yukiyasu Kamitani (kamitani@i.kyoto-u.ac.jp).

#### Materials availability

This study did not generate new unique reagents.

### Experimental model and subject details

#### Deep neural networks

We compared 29 pre-trained DNNs in this study (Table 1). All DNNs were pre-trained on ImageNet ILSVRC 2012 dataset (Deng et al., 2009) to classify given images into 1000 object categories. We used AlexNet (Krizhevsky et al., 2012), VGG-16, VGG-19 (Simonyan and Zisserman, 2014) , VGG-S, VGG-M, VGG-F (Chatfield et al., 2014), ResNet-18, ResNet-34 (He et al., 2015), ResNet-50-v2, ResNet-101-v2, ResNet-152-v2 (He et al., 2015), Inception-v1, Inception-v2, Inception-v3, Inception-v4 (Deco et al., 2015; Szegedy et al., 2016, 2014) , Inception-ResNet-v2 (Szegedy et al., 2016), SqueezeNet-1.0, SqueezeNet-1.1 (Iandola et al., 2016), DenseNet-121, DenseNet-161, DenseNet-169, DenseNet-201 (Huang et al., 2016), NASNet-Mobile, NASNet-Large (Zoph et al., 2018), PNASNet-Large (Liu et al., 2018), MobileNet-v2-1.4-224 (Sandler et al., 2019), CORnet-Z, CORnet-R, and CORnet-S (Kubilius et al., 2018) .

To characterize hierarchy in DNNs, we included several representative layers of each DNN in the analysis: the first layer, the last fully-connected (FC) layer (referred to as the “category layer” in this study), the other FC layers, all convolutional layers in DNNs without submodules (i.e., AlexNet, VGG-S, VGG-M, and VGG-F), and the output layers of submodules (i.e., convolutional, residual, or inception blocks) in DNNs with submodules. For residual blocks, we regarded the sum of skip layers as the output of the block. Hereafter, “layer” means the representative layers unless otherwise stated. As CORnet-R and CORnet-S have recurrent connections, we used outputs of the last time-step of these DNNs.

### Method details

#### fMRI dataset

We used the functional magnetic resonance imaging (fMRI) dataset collected in Shen et al. (2019). The dataset, which is publicly available at OpenNeuro (https://openneuro.org/datasets/ds001506), includes fMRI data from three human subjects. During fMRI scanning sessions, subjects viewed natural object images selected from ImageNet (2011, fall release, Deng et al., 2009). The fMRI experiment was composed of two types of sessions: training and test sessions. In training sessions, 1200 images selected from 150 categories were used. Each image was presented five times. In test sessions, 50 images selected from 50 categories were presented. Each image was presented 24 times. During the experiment, subjects performed one-back repetition tasks. Voxel size was 2 × 2 × 2 mm, and TR was 2 s in the fMRI measurement. The fMRI responses in the training and test sessions were used for training and test of decoders/encoders, respectively (see Decoding analysis” and “Encoding analysis). The fMRI responses in test sessions were also used for the representational similarity analysis in Figure S9.

Motion correction and anatomical-functional coregistration to individual brains were performed on the fMRI signals with SPM (http://www.fil.ion.ucl.ac.uk/spm). After preprocessing, nuisance parameters (head motion parameters and linear trend) were regressed out from the signal of each voxel. Then, the signal amplitudes were normalized relative to the mean amplitude during the initial rest period (24 s) of each run, and despiked by reducing extreme values (beyond ±3 standard deviations in each run). The signal time series were shifted by 4 s to compensate for hemodynamic delays. The fMRI response to each image was obtained by averaging fMRI signals during the presentation block (8 s) of each image.

#### Region of interest

Five visual areas (V1, V2, V3, V4, and higher visual cortex [HVC]) were included in the analysis. All regions of interest (ROIs) were defined functionally on individual brains. V1, V2, V3, and V4 were delineated based on standard retinotopy experiments (Engel et al., 1994; Sereno et al., 1995). The HVC was manually delineated as a contiguous region covering the lateral occipital complex (LOC), the fusiform face area (FFA), and the parahippocampal place area (PPA). The LOC, FFA, and PPA were identified by the conventional functional localizer experiments (Epstein and Kanwisher, 1998; Kanwisher et al., 1997; Kourtzi and Kanwisher, 2000).

In a complementary analysis, we divided the HVC into three subregions based on the principal gradient (Margulies et al., 2016). We aligned principal gradient maps into the individual brain space. Then, one-third of voxels in HVC having the lowest, middle, or highest values of Gradient 1, which presumably corresponds to unimodal-transmodal axis, were grouped as HVC-1, 2, and 3, respectively. We also divided the visual cortex into either 5 or 10 ROIs based on Gradient 1 of the principal gradient.

#### Decoding analysis

For each individual unit in a DNN, we constructed a decoder to predict (decode) the unit activation to an image from fMRI response patterns to the same image. We used linear regression with L2-regularization to construct the decoders. The input feature of the decoder was an fMRI response pattern of 500 voxels in one of the five ROIs. We selected the 500 voxels that showed the highest absolute correlations between their fMRI signals and the target unit activations in the training sessions (Shen et al., 2019). The unit activations in the first layers of DNNs were converted into absolute values, since both increments and decrements of stimulus luminance strongly modulate fMRI signals in the early visual cortex (Haynes et al., 2004) and the absolute values of unit activations in the first layer are better predicted than raw values (Shen et al., 2019). The decoders were trained with fMRI data in the training sessions for each subject. After the training of decoders, we predicted activations of the individual units from fMRI response patterns in the test sessions. In the test fMRI data, responses of individual voxels to the same images were averaged across trials to increase the signal-to-noise ratio of the fMRI signals. Thus, we obtained for each DNN unit 50 predicted activation values corresponding to 50 images in the test sessions. The prediction accuracy was evaluated as the Pearson correlation coefficient between the actual and predicted unit activations across the test images. For each DNN layer, activations of randomly selected 1000 units were predicted if the number of units was more than 1000. Otherwise, activations of all units were predicted. We ran decoding of individual DNN unit activations using fMRI response patterns from five ROIs in three subjects independently.

#### Encoding analysis

For individual voxels in the ROIs, we constructed an encoding model to predict the voxel response to an image from unit activation patterns in a DNN layer to the same image. We used linear regression with L2-regularization to construct the encoding models. For each layer, activations of 500 units were selected in the same procedure as decoding analysis. As in decoding analysis, the unit activations in the first layer of DNNs were converted into absolute values. The encoding models were trained with fMRI data in the training sessions for each subject. After the training of encoding models, we predicted responses of the individual voxels from DNN unit activation patterns to the images in the test sessions. Thus, for each voxel, we obtained 50 predicted response values corresponding to 50 images in the test sessions. In the test fMRI data, the responses of individual voxels to the same images were averaged across trials. The prediction accuracy was evaluated as the Pearson correlation coefficient between the predicted and observed responses of voxels across the test images.

In the replication analysis of Schrimpf et al. (2018), the median of encoding accuracies across voxels in V4 and HVC was computed for each DNN layer. The encoding accuracies were obtained for fMRI data of each subject, then averaged across subjects. For a given DNN, the highest encoding accuracy for V4 among the DNN layers was defined as “fMRI V4 encoding accuracy” and the highest mean encoding accuracy for HVC among the DNN layers was defined as “fMRI HVC encoding accuracy.”

#### Brain hierarchy (BH) score

The decoding- and encoding-based brain hierarchy (BH) scores were computed with the results of the decoding and encoding analyses, respectively. Their mean was reported as the BH score. For calculation of the BH score, the ROIs were assigned numbers as 1 to 5 from the lower to the higher visual areas (i.e., V1 (1), V2 (2), V3 (3), V4 (4), HVC (5)). Similarly, the DNN layers in each DNN were assigned numbers from1 to N in order from input to output (N is the number of layers in the DNN).

The decoding-based BH score was calculated for each DNN using the following procedure. We randomly selected three layers from the representative layers except the first and last (category) layers. The five layers (the selected three layers, the first layer, and the last layers) were included in the calculation of the BH score. The layer selection and subsequent calculation of the decoding-based BH score was repeated 10,000 times for each DNN. For each unit in the selected layers, we identified the ROI that had the highest prediction accuracy (“top ROI”) based on the decoding analysis. Then, we applied an optional unit selection; units in which prediction accuracy of the top ROI was not significantly higher than the chance level (*t*-test, *p* < 0.05, uncorrected) were excluded from the further analysis. The remaining units were pooled across the DNN layers and subjects. The decoding-based BH score of a DNN was defined as the Spearman rank correlation coefficient between the layer number and the top ROI number across units in the DNN.

The encoding-based BH score was computed for each DNN by swapping units and voxels in the calculation of the decoding-based BH score. Unlike the decoding-based BH score, all representative layers were included in the calculation of the encoding-based BH score. Based on the encoding analysis, we identified for each voxel the layer that had the highest prediction accuracy (“top layer”). We then applied optional voxel selection: voxels in which the prediction accuracy of the top layer was not significantly higher than the chance level (*t-*test, *p* < 0.05, uncorrected) were excluded from the further analysis. The survived voxels were pooled across the ROIs and subjects. The encoding-based BH score of a DNN was defined as the Spearman rank correlation coefficient between the ROI number and the top layer number across voxels.

The brain-to-brain BH score was computed by treating the brain of one target subject as a 5-layer DNN, in which we regarded ROIs (V1, V2, V3, V4, and HVC) as DNN layers, and regarded fMRI voxels as DNN units. The activity of each voxel of the target subject's brain was predicted from brain activities in each ROI of two other subjects' brains. As with the original BH score, we selected the ROI with the highest prediction accuracy (top ROI) for each voxel in the target brain. Then, we excluded voxels in the target brain when their prediction accuracy from the top ROI was not significant (*t*test, *p* < 0.05, uncorrected). We defined the brain-to-brain BH score as the Spearman rank correlation coefficient between the ROI number and top ROI number across voxels in the target brain. We repeat this computation by changing the target subject within three subjects. The mean brain-to-brain BH score across three subjects was reported in Figure 3.

#### Manipulation of DNN architecture

We manipulated the number of FC layers in AlexNet (Krizhevsky et al., 2012) by replacing the layers with convolutional or FC layers. The default AlexNet had five convolutional, two FC, and one category layer. All and the first FC layers were replaced with convolutional layers to create a DNN with zero and one FC layer, respectively. The last one, two, and three convolutional layers were replaced with FC layers to create a DNN with three, four, and five FC layers. DNNs with 0, 1, 2, 3, 4, and 5 FC layers achieved 0.55, 0.57, 0.56, 0.51, and 0.46 ImageNet top-1 accuracies, respectively.

To manipulate kernel sizes, we modified a DNN with six convolutional and one category layer. The kernel sizes of the convolutional layers were changed from 1 × 1 to 6 × 6, where the 6 × 6 kernel is equivalent to FC layers. DNNs with kernel size 1 × 1, 2 × 2, 3 × 3, 4 × 4, 5 × 5, and 6 × 6 achieved 0.53, 0.53, 0.56, 0.57, 0.57, and 0.57 ImageNet top-1 accuracies, respectively.

The presence of skip-connections was manipulated by replacing all convolutional layers in AlexNet with residual blocks of ResNet-18 (He et al., 2015). The model with skip-connections achieved 0.64 ImageNet top-1 accuracy. Similarly, the presence of branch-connection was manipulated by replacing all convolutional layers in AlexNet with inception blocks of Inception-v1 (Szegedy et al., 2014). The model with branch-connections achieved 0.58 ImageNet top-1 accuracy.

To manipulate the depth of a DNN, we inserted or removed additional convolutional layers into/from VGG-19 (Simonyan and Zisserman, 2014). Note that the additional convolutional layers were not included in the calculation of BH scores (i.e., the insertion of additional layers did not change the layer numbers). The default VGG-19 had a depth of 19 (16 convolutional, two FC layers, and one category layer). The depth was reduced to eight by removing convolutional layers in each of five convolutional blocks except the representative layers. The depth was increased to 37 and 52 by inserting additional convolutional layers in each convolutional block: 0, 6, 4, 4, and 4 layers were inserted in the first to fifth convolutional blocks for depth of 37, and 5, 8, 8, 6, and 6 layers were inserted in the first to fifth convolutional blocks for depth of 52. These models achieved 0.58, 0.72, 0.63, and 0.68 ImageNet top-1 accuracies, respectively.

All DNNs were trained with an image category classification task on the ImageNet ILSVRC 2012 dataset. The batch size was 64 and the learning rate was 0.01, which was multiplied by 0.1 for every 20 epochs. The cost function was cross-entropy with L2 penalty. The coefficient of the L2 penalty term was 5 × 10ˆ(-4). DNN weights were optimized by gradient descent with momentum (Qian, 1999) with a momentum term of 0.9. Dropout operations were inserted into every fully-connected layer except for the last layer. The dropout rate was set to 0.5. To prevent serious overfitting, we utilized early stopping based on the validation set.

#### Feature importance analysis

We computed the proportion of input features (DNN unit or fMRI voxel) that contributed to prediction of at least one of target features. Firstly, we computed correlation coefficients between each pair of target feature and input feature. For each target feature, we ranked input features with the absolute value of the correlation coefficients between the target feature and each input feature. We then performed a decoding/encoding analysis while adding the input features one by one in order of the input features' ranking, and computed the prediction accuracy. We terminated this operation when the prediction accuracy reached 95% of that when all input features were used, and obtained the index of input features that were not used for the prediction. We repeated this procedure for randomly selected 1000 target features, and took the intersection (AND) of the indies for all target features. The input features included in the intersection did not contribute to the prediction of any target features. The proportion of input features contributing to prediction was obtained by 1 – the proportion of the non-contributing features.

### Quantification and statistical analysis

A permutation test was applied to examine whether the Spearman rank correlation coefficient between the BH score and image recognition performance is significantly deviated from zero (Figure 3). The tail probabilities of Spearman's rho were computed using the Edgeworth series approximation (David et al., 1951).

## Data Availability

• We used the fMRI data collected from Shen et al. (2019). The data are available in public repositories: raw fMRI data are hosted at OpenNeuro (https://openneuro.org/datasets/ds001506) and preprocessed fMRI data are provided at figshare (https://doi.org/10.6084/m9.figshare.7033577). The unit activations of the DNNs as well as the decoded unit activations and the prediction accuracies are available at figshare (https://doi.org/10.6084/m9.figshare.12401168). The stimulus images used in the fMRI experiment by Shen et al. (2019) are available on request (https://forms.gle/ujvA34948Xg49jdn9). • The code to reproduce the results in this study is available at GitHub (https://github.com/KamitaniLab/BHscore). The BH scores can be calculated by a function in our code repository. • Any additional information required to reanalyze the data reported in this paper is available from the lead contact upon request.
