# Supplementary material for: Brain hierarchy score: Which deep neural networks are hierarchically brain-like?
Source: iScience. 2021 Aug 21;24(9):103013. doi: 10.1016/j.isci.2021.103013 (PMC8426272; doi:10.1016/j.isci.2021.103013)
Supplement: Document S1. Figures S1–S10 [file mmc1.pdf]

iScience, Volume *24*

Supplemental information

Brain hierarchy score: Which deep neural networks are hierarchically brain-like?

Soma Nonaka, Kei Majima, Shuntaro C. Aoki, and Yukiyasu Kamitani

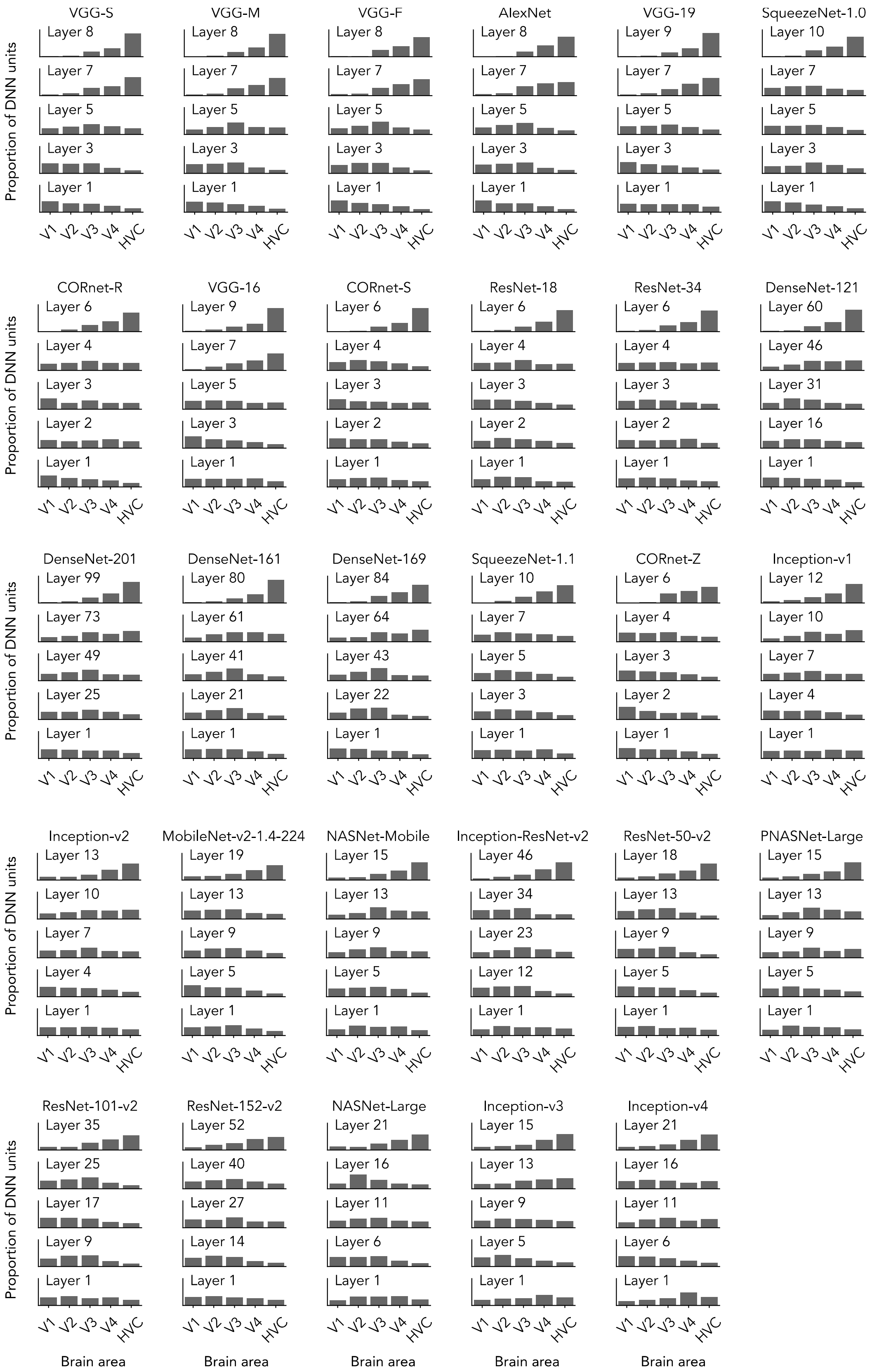

Figure S1. Top ROI distributions for all 29 deep neural networks (DNNs), Related to Figure 2. The

distributions of top ROIs for individual layers of 29 DNNs are shown. DNNs were sorted in the order of their decoding-based brain hierarchy (BH) scores. For visualization purposes, the results for five layers sampled from each DNN are plotted.

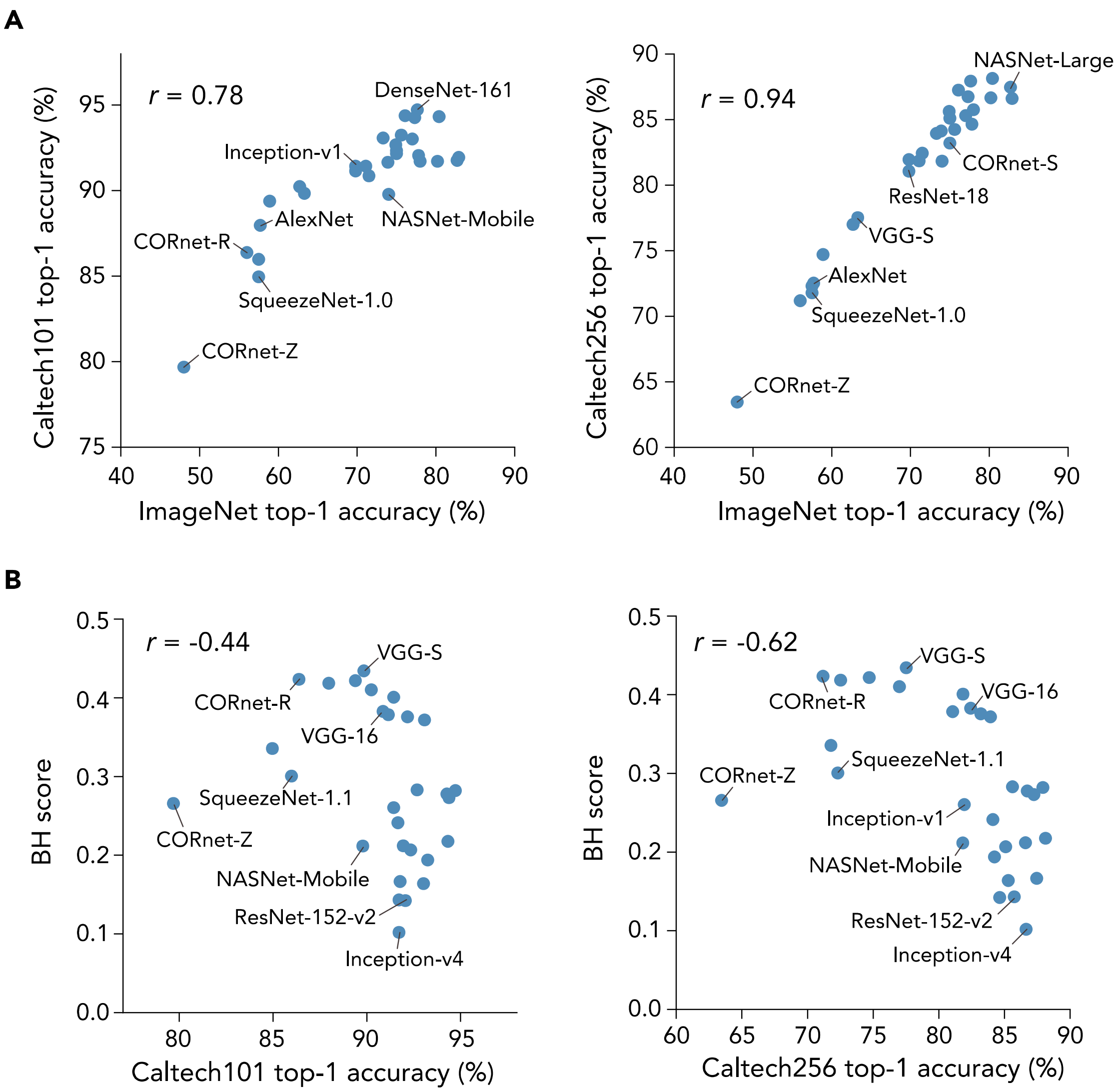

Figure S2. Image recognition performance and BH scores of DNNs evaluated using different image datasets, Related to Figure 3. (A) Image recognition performance. ImageNet top-1 accuracy is plotted against the image recognition accuracy evaluated on Caltech-101 (left) and Caltech-256 (right). To evaluate the accuracy on Caltech-101 and Caltech-256, we trained a multinomial logistic regression classifier using the second last layer of each DNN as input. **(B)** The BH scores and image recognition accuracies on Caltech-101 and Caltech-256. The BH scores for 29 DNNs are plotted against the image recognition accuracies evaluated on Caltech-101 and Caltech-256.

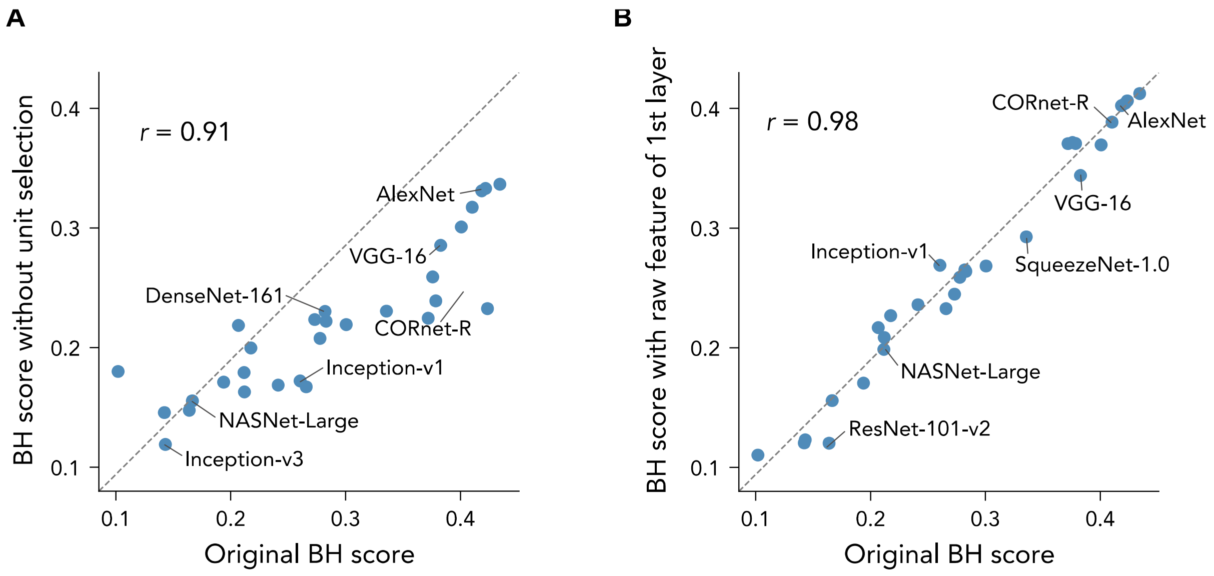

Figure S3. Robustness of BH scores on computation procedures, Related to Figure 3. (A) BH scores computed with and without unit selection. BH scores computed without unit selection are plotted against BH scores computed with our original definition of the BH score. **(B)** BH scores computed with raw features of the earliest layer and with our original definition of the BH score. BH scores computed with raw features of the earliest layer are plotted against BH scores computed with our original definition of the BH score.

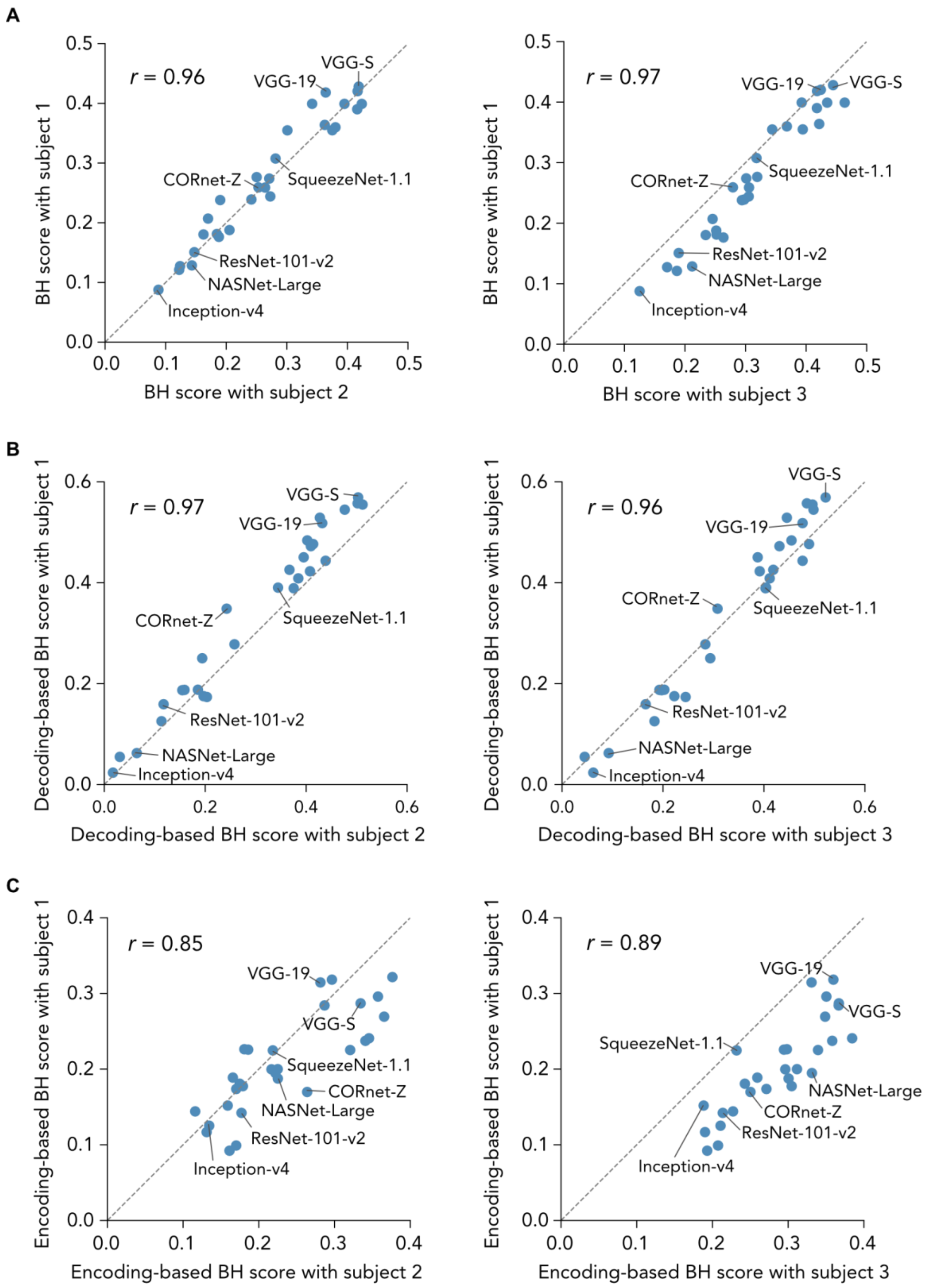

Figure S4. Consistency of the BH scores across fMRI data from different subjects, Related to Figure 3. (A) Consistency of BH scores between subjects. To confirm the robustness of BH scores, we computed the scores from fMRI data of each single subject. BH scores computed from the functional

magnetic resonance imaging (fMRI) data of one subject are plotted against BH scores computed from the fMRI data of other subjects. **(B)** Consistency of decoding-based BH scores between subjects. **(C)** Consistency of encoding-based BH scores between subjects.

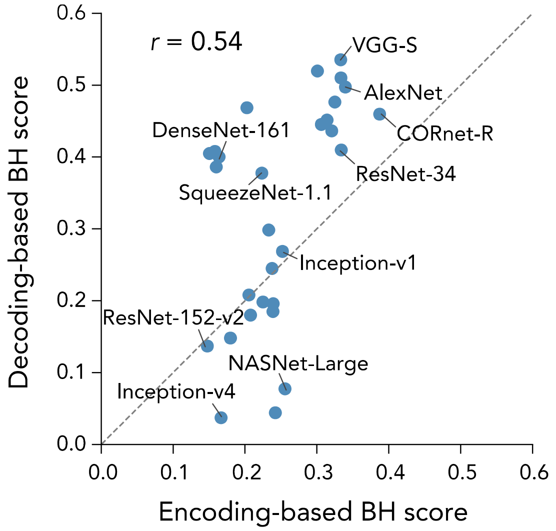

Figure S5. Consistency between BH scores obtained by decoding analysis and BH scores obtained by encoding analysis, Related to Figure 3. BH scores obtained by decoding analysis and by encoding analysis. Encoding analysis was performed where the fMRI responses of individual voxels were predicted from the responses of each DNN layer. For each fMRI voxel, the DNN layer showing the highest prediction accuracy (best layer) was assigned.

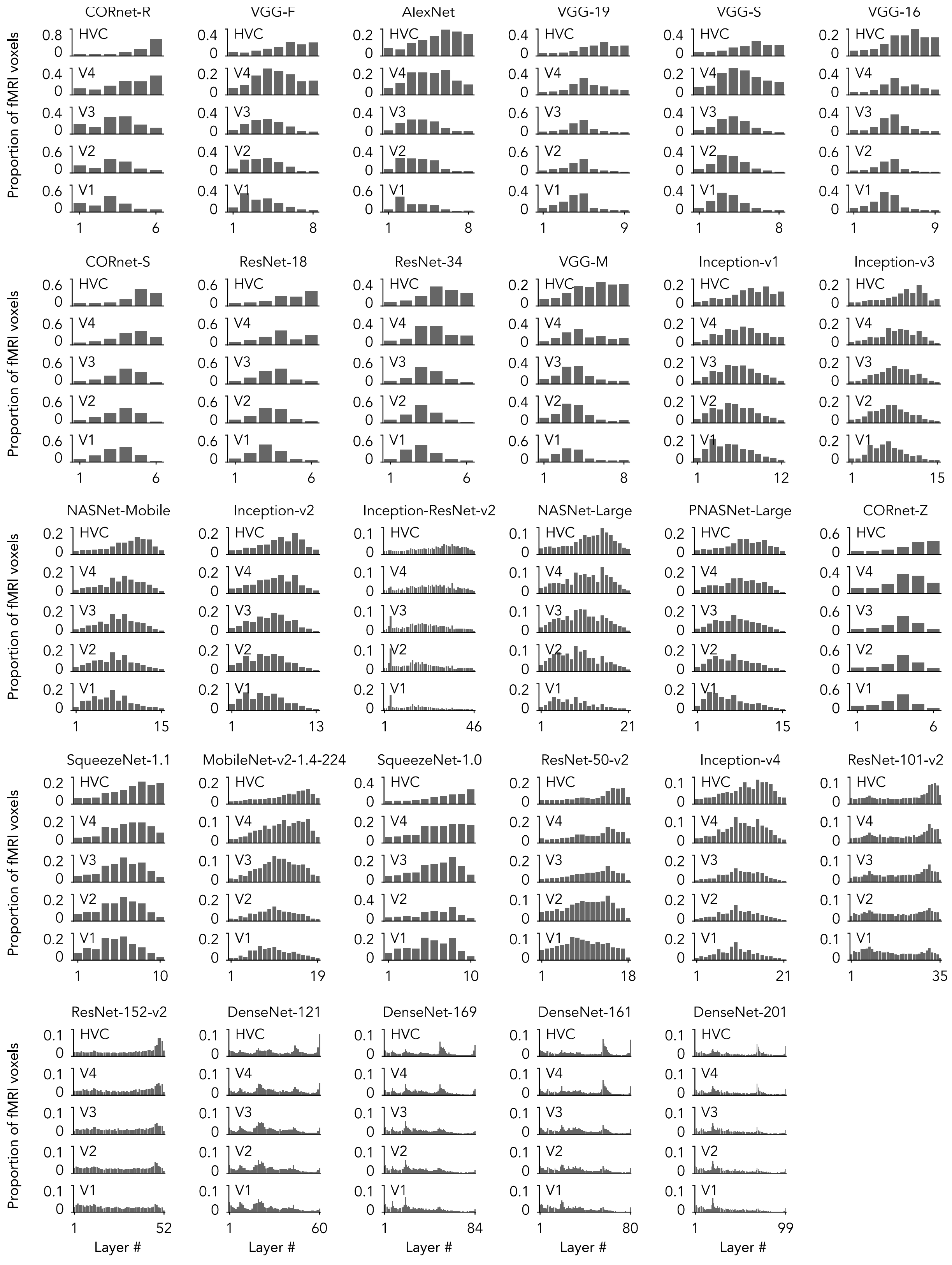

Figure S6. Distributions of top DNN layers for all 29 DNNs, Related to Figure 3. In the encoding analysis, fMRI responses of individual voxels in each ROI were predicted from the DNN unit responses in

each DNN layer of 29 DNNs. Then, for each voxel, the DNN layer showing the highest prediction accuracy among the layers in each DNN (top layer) was identified. The distribution of the best layers for voxels in each ROI is shown. DNNs were sorted in the order of their encoding-based BH scores. The range of the y-axis differs across DNNs and layers for visualization purposes.

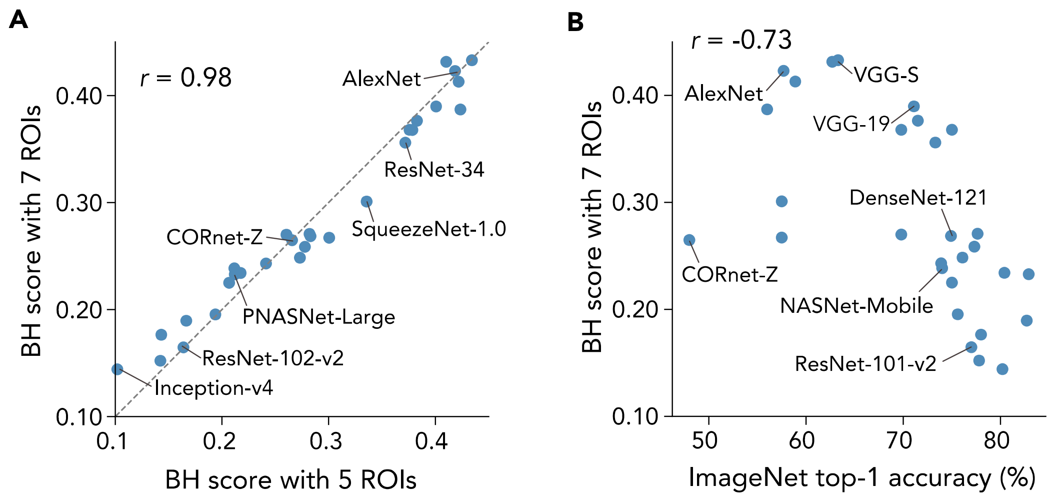

Figure S7. Consistency of the BH scores across different ROI parcellations, Related to Figure 3. **(A)** BH scores obtained with 5 and 7 ROIs. To examine whether finer parcellation of ROIs affects BH scores, the BH scores calculated with 7 ROIs (V1, V2, V3, V4, HVC-1, HVC-2, and HVC-3) were compared to the BH scored calculated with 5 ROIs (V1, V2, V3, V4, and HVC). The HVC was divided into three regions based on the principal gradient (Margulies et al., 2016). **(B)** BH scores with 7 ROIs versus ImageNet top-1 accuracies. The BH scores calculated with the 7 ROIs were plotted against the ImageNet top-1 accuracy of each DNN.

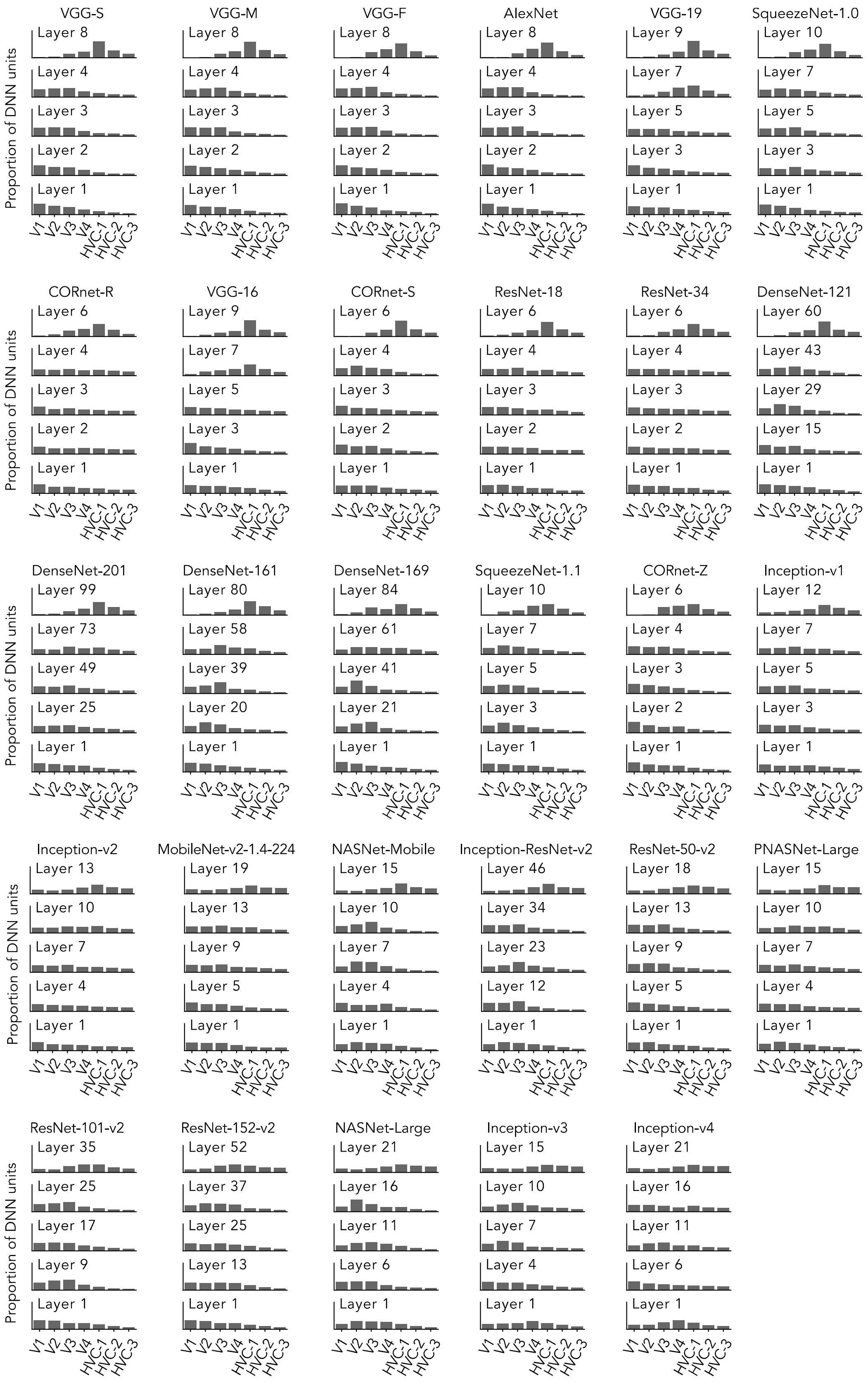

Figure S8. Distributions of top ROIs across 7 ROIs for all 29 DNNs with 7 ROIs, Related to Figure

3. The distributions of top ROIs across 7 ROIs (V1, V2, V3, V4, HVC-1, HVC-2, and HVC-3) for individual layers of 29 DNNs are shown. For visualization purposes, the results for five layers sampled at even intervals are plotted for each DNN.

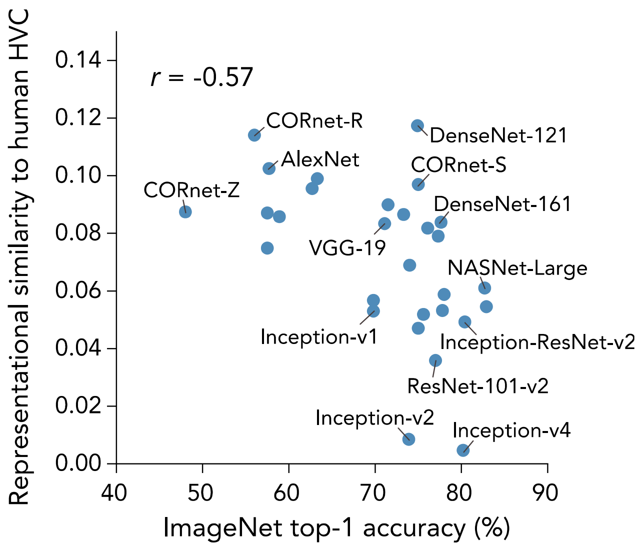

Figure S9. Representational similarity to human higher visual cortex (HVC) and ImageNet top-1 accuracy, Related to Figure 3. The representational similarities between DNNs and the human HVC were quantified by following the procedure reported by Jozwik et al. (2019), and those are plotted against ImageNet top-1 accuracies.

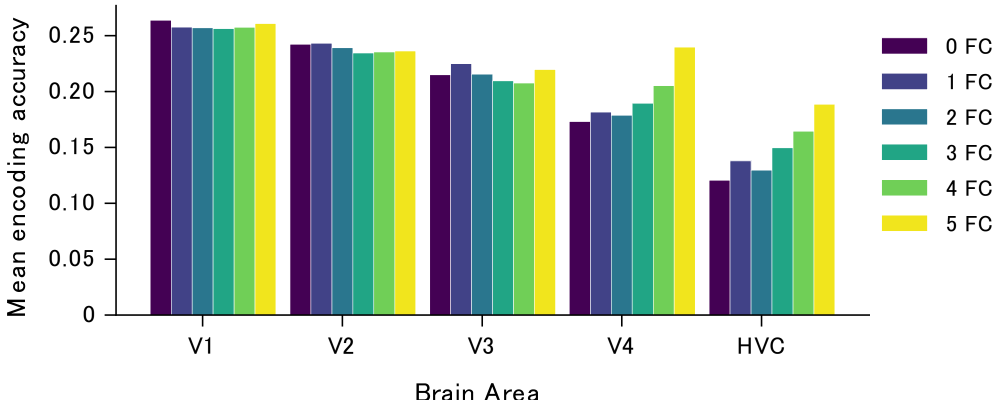

Figure S10. Comparison of encoding accuracies by DNNs with different numbers of FC layers, Related to Figure 8. We performed an encoding analysis by following the procedure of Schrimpf et al. (2018). The mean encoding accuracies for 5 brain areas obtained by DNNs with different numbers of FC layers are shown.
